# Structural Characterization of the RNA-Binding Protein SERBP1 Reveals Intrinsic Disorder and Atypical RNA Binding Modes

**DOI:** 10.3389/fmolb.2021.744707

**Published:** 2021-09-24

**Authors:** Antoine Baudin, Alma K. Moreno-Romero, Xiaoping Xu, Emily E. Selig, Luiz O. F. Penalva, David S. Libich

**Affiliations:** ^1^ Greehey Children’s Cancer Research Institute, The University of Texas Health Science Center at San Antonio, San Antonio, TX, United States; ^2^ Department of Biochemistry and Structural Biology, The University of Texas Health Science Center at San Antonio, San Antonio, TX, United States; ^3^ Department of Cell Systems and Anatomy, The University of Texas Health Science Center at San Antonio, San Antonio, TX, United States

**Keywords:** SERBP1, RNA binding protein, mRNA binding, intrinsically disordered protein, NMR

## Abstract

RNA binding proteins (RBPs) are essential for critical biological processes such as translation regulation and mRNA processing, and misfunctions of these proteins are associated with diseases such as cancer and neurodegeneration. SERBP1 (SERPINE1 mRNA Binding Protein 1) is an RBP that comprises two RG/RGG repeat regions yet lacks other recognizable RNA-binding motifs. It is involved in mRNA maturation, and translational regulation. It was initially identified as a hyaluronic acid binding protein, but recent studies have identified central roles for SERBP1 in brain function and development, especially neurogenesis and synaptogenesis. SERBP1 regulates One-carbon metabolism and epigenetic modification of histones, and increased SERBP1 expression in cancers such as leukemia, ovarian, prostate, liver and glioblastoma is correlated with poor patient outcomes. Despite these important regulatory roles for SERBP1, little is known about its structural and dynamic properties, nor about the molecular mechanisms governing its interaction with mRNA. Here, we define SERBP1 as an intrinsically disordered protein, containing highly conserved elements that were shown to be functionally important. The RNA binding activity of SERBP1 was explored using solution NMR and other biophysical techniques. The outcome of these experiments revealed that SERBP1 preferentially samples compact conformations including a central, stable α-helix and show that SERBP1 recognizes G-rich RNA sequences at the C-terminus involving the RGG box and neighboring residues. Despite the role in RNA recognition, the RGG boxes do not seem to stabilize the central helix and the central helix does not participate in RNA binding. Further, SERBP1 undergoes liquid-liquid phase separation, mediated by salt and RNA, and both RGG boxes are necessary for the efficient formation of condensed phases. Together, these results provide a foundation for understanding the molecular mechanisms of SERBP1 functions in physiological and pathological processes.

## Introduction

SERBP1 (SERPINE1 mRNA binding protein 1) is a highly conserved RNA binding protein (RBP) containing two RG/RGG repeat regions yet lacks other readily recognizable, canonical or structured RNA binding motifs. RBPs containing RG/RGG repeats are essential for normal brain function and have been implicated in neurological and neuromuscular diseases as well as certain cancers (Järvelin et al., 2016; Hentze et al., 2018). High SERBP1 expression in glioblastoma multiform (GBM) is linked to poor patient outcome and response to therapy while *in vitro* and *in vivo* studies showed that expression levels of SERBP1 affect several related cancer phenotypes, stemness, neuronal differentiation and tumor growth (Koensgen et al., 2007; Serce et al., 2012; Costa et al., 2014; Wang et al., 2017; Kosti et al., 2020). We recently established that SERBP1 functions as a novel oncogenic factor in GBM through regulation of One-carbon metabolism, methionine production, and histone methylation (Kosti et al., 2020). Moreover, knockdown of SERBP1 affected the expression of genes linked to neurogenesis and synaptogenesis. A strong negative expression correlation was observed between genes in these categories and SERBP1 both in brain and patient-derived GBM samples, implicating SERBP1 in brain function and development (Kosti et al., 2020). Two proximity-dependent biotinylating screening studies identified SERBP1 as an interaction partner of RBPs known to regulate synaptic plasticity such as FMR1, FXR1, FXR2, CAPRIN1, and SYNCRIP (Youn et al., 2018; Go et al., 2021). Additionally, SERBP1 has a role in the SUMOylation of certain proteins (Lemos and Kobarg, 2006), and is itself SUMOylated (Hendriks et al., 2014) on a lysine-rich sequence between the two RGG boxes. SUMOylation of SERBP1 has been suggested as a factor in the development of GBM, as aberrations in SUMOylation pathways can lead to the development of cancer (Fox et al., 2019).

SERBP1 structure and its RNA recognition and binding activity are poorly characterized. SERBP1 was reported to bind preferentially to GC-rich motifs (Kosti et al., 2020), subsequent studies revealed that these motifs could include G-quadruplexes (Su et al., 2021). SERBP1 was identified in the structures of non-translating 80 S ribosomes, blocking the mRNA entrance channel suggesting that it serves to regulate mRNA translation (Ahn et al., 2015; Brown et al., 2018; Muto et al., 2018). Like SERBP1, the SARS-CoV-2 non-structural protein 1 (Nsp1) mediates translation inhibition of mRNA by binding and blocking the ribosomal mRNA channel through interactions with its disordered C-terminal domain (Schubert et al., 2020). Translational regulation does not account for the RNA binding activity of SERBP1 and thus it likely participates in additional regulatory processes. For instance, vig and vig2, SERBP1 Drosophila homologues, have been identified in RNAi complexes and heterochromatin, (Gracheva et al., 2009), and as regulators of histone genes (Tsui et al., 2018). As noted above, SERBP1 also interacts with arginine-methylated and stress granule-associated proteins (Youn et al., 2018), and selective methylation of either RGG repeat region modulates its subcellular distribution between the nucleus and cytoplasm (Lee et al., 2012a). To better understand the structural determinants guiding SERBP1 roles in these diverse physiological and pathological processes, a structural and biophysical analysis was undertaken to characterize its structural and functional properties. A combination of solution NMR spectroscopy and biophysical assays were used to define the structural and dynamic properties of SERBP1. The data reveal that SERBP1 is primarily an intrinsically disordered protein (IDP) yet it adopts compact, and partially structured conformations. Moreover, the SERBP1 binding site of a GC-rich RNA oligonucleotide was identified, and it was determined that RNA binding negatively affects the liquid-liquid phase separation (LLPS) propensity of SERBP1. Together, these results are a step toward understanding the multifunctional nature of SERBP1 and determining the structural underpinnings of its diverse physiological roles in healthy cells as well as its aberrant function in GBM and other tumor types.

## Methods

### Phylogenetics

COBALT was used to perform multiple sequence alignment to identify conserved regions among SERBP1 homologues from eleven different species (nine different phyla) (Papadopoulos and Agarwala, 2007). Amino-acid identity was prioritized, and an amino-acid was defined as conserved if it was present in at least seven species. Protein sequences were obtained from the NCBI (https://www.ncbi.nlm.nih.gov/) with the following accession numbers: *H. sapiens* (NP_001018077.1), *Saccoglossus kowalevskii* (XP_002740974.1), *Acanthaster planci* (XP_022107686.1), *Drosophila melanogaster* (NP_523572.1), *Stegodyphus mimosarum* (KFM69747.1), *Centruroides sculpturatus* (XP_023216060.1), *Octopus sinensis* (XP_029640355.1), *Teladorsagia circumcincta* (PIO76243.1), *Echinococcus granulosus* (XP_024349465.1), *Nematostella vectensis* (XP_001637231.1), *A. queenslandica* (XP_011406826.2).

### Protein Expression and Purification

The SERBP1 189–400 construct (Addgene accession number 172315) was expressed in *E.coli* as previously described (Baudin et al., 2021). The genes for full-length SERBP1 and a SERBP1 149-400 construct were amplified by PCR (primer are shown in Supplementary Table S1), digested by *Kas*I and *Bam*HI restriction enzymes and cloned into a custom pAG8Ha-His vector that introduced an 8x histidine tag followed by a TEV cleavage site N-terminal to the coding sequence. Plasmids were transformed by heat shock into *E. coli* BL21 Star™ (DE3) (Invitrogen, MA) and a colony from the resultant agar plate was used to inoculate a 5 ml LB starter culture that was grown at 37^°^C for 6–8 h. The LB starter culture was used to inoculate a 100 ml M9 preculture that was grown overnight at 37^°^C with shaking. The overnight preculture was then used to inoculate an expression culture of 900 ml M9 minimal media. Both the 100 ml starter and 900 ml expression cultures were supplemented with ^15^NH_4_Cl (^13^C-glucose) for isotopic enrichment. For non-isotopically enriched protein, the overnight culture was 10 ml LB and 4 ml was used to inoculate a 1 L LB expression culture. All cultures were supplemented with 100 μg/ ml of ampicillin. Expression cultures were grown at 37°C in baffled Fernbach flasks with shaking, and protein expression was induced at OD_600_ ∼0.6–0.8 with 1 mM IPTG and continued for 3 or 6 h, for LB or M9 cultures, respectively. The cells were harvested by centrifugation at 4000 g for 20 min and the resulting pellets were stored at −80°C.

SERBP1 constructs were purified as previously described for SERBP1 189–400 (Baudin et al., 2021). Briefly, frozen *E. coli* pellets were thawed and resuspended in 8 M urea, 50 mM Tris pH 8.0, 150 mM NaCl, 20 mM imidazole, lysed by sonication (6 cycles of 10 s on, 30 s off), and the lysate was cleared by centrifugation for 30 min at 45000 g at 4°C. The supernatant was applied to a 5 ml HisTrap HP column (Cytiva, MA, United States), equilibrated with the lysis buffer, washed with 25 column volumes of the same buffer, and eluted with 8 M urea, 50 mM Tris pH 8.0, 150 mM NaCl, 500 mM imidazole. Eluted protein was concentrated with a 3 kDa (149–400, 189–400) or 10 kDa (full-length) cutoff Amicon centrifugal concentrator (Merck, NJ, United States) to ∼4 ml and diluted into 40 ml of 100 mM sodium phosphate buffer, pH 7, 1 mM PMSF, 1 mM EDTA buffer. Precipitated protein was removed by centrifugation at 3,000 g for 15 min, and the protein was then dialyzed against 100 mM sodium phosphate buffer pH 7 (3 x 2 L), on the third change 500 µL of 1.6 mg/ ml Tobacco Etch Virus (TEV) protease was added and the mixture was incubated at room temperature overnight. The 8x His tag was not removed from full-length SERBP1. Post-dialysis, the sample was centrifugated at 3,000 g for 15 min to remove any precipitate and concentrated to approximately 4 ml with an Amicon centrifugal concentrator. A final cation exchange chromatography polishing step was used for SERBP1 149–400 and 189–400. The 4 ml sample was diluted into 60 ml with 50 mM sodium acetate buffer pH 4.5, 0.5 mM PMSF, 0.5 mM EDTA, loaded on a 5 ml SP Sepharose fast-flow column (Cytiva, MA, United States), washed with 20 column volumes of the same buffer and eluted with a 0.05–1 M NaCl gradient. Fractions containing SERBP 149–400 were concentrated, and buffer exchanged into NMR buffer (20 mM sodium phosphate buffer pH 6.9, 60 mM NaCl, 1 mM PMSF, 0.2 mM EDTA, 10 % D_2_O).

### NMR Spectroscopy

All experiments were recorded on a Bruker Avance NEO spectrometer operating at a proton Larmor frequency of 700.13 MHz, at a temperature of 5°C using a 5 mm TCI *z*-axis gradient cryogenic probe. Data were processed with the NMRPipe software suite (Delaglio et al., 1995) and analyzed with CCPNMR Analysis 2.5 software (Skinner et al., 2016). SERBP1 149–400 ^1^H, ^13^C_α_, ^13^C_β_, ^13^C’ and ^15^N backbone resonances were assigned through the analysis of a set of 2D and 3D experiments, namely ^1^H,^15^N-HSQC, HNCACB, CBCA(CO)NH, HNCO, HN(CA)CO and HCC(CO)NH, recorded on a ^13^C,^15^N-labeled sample at a concentration of 300 μM, in NMR buffer. The ^1^H,^15^N-HSQC was recorded with 128*x 1024* complex points in the indirect (^15^N) and direct (^1^H) dimensions, corresponding to acquisition times of 75.2 and 112.6 ms, respectively. Acquisition parameters for the HNCO and HN(CA)CO consisted of 32^∗^ x 64^∗^ x 1024^∗^ complex points in the indirect (F1, ^13^C), (F2, ^15^N) and direct (F3 ^1^H) dimensions, corresponding to acquisition times of 16.5, 37.6, 112.6 ms, respectively; acquisition parameters for the HNCACB, CBCA(CO)NH and HCC(CO)NH consisted of 128^∗^ x 64^∗^ x 1024^∗^ complex points in the indirect (F1, ^13^C), (F2, ^15^N) and direct (F3 ^1^H) dimensions, corresponding to acquisition times of 11, 37.6, 112.6 ms, respectively. All 3D experiments were recorded in non-uniform sampling (NUS) mode (Delaglio et al., 2017) with a sampling density of 20%, and the spectra were reconstructed using the SMILE algorithm implemented in NMRPipe (Ying et al., 2017).

^15^N *R*_1_ and *R*_2_ relaxation rates were calculated from *T*_1_ and *T*_1*ρ*_ experiments, recorded on 50 and 100 µM samples for SERBP1 189–400 and SERBP1 149–400, respectively, in NMR buffer using 64* x 1024* complex data points in the indirect (^15^N) and direct (^1^H) dimensions corresponding to acquisition times of 37.6 and 112.6 ms, respectively. The ^15^N *T*_1_ experiment consisted of eight interleaved spectra with the following relaxation delays: 40, 80, 200, 280, 300, 400, 600, and 800 ms. The *T*_1*ρ*_ experiment was recorded using a *B*_1_ field of 1400 Hz and eight interleaved spectra with the following relaxation delays: 1, 21, 31, 41, 61, 81, 121 and 161 ms. ^15^N *R*_2_ rates were calculated using the following equation (Massi et al., 2004):

$$R_{1\rho }=R_{1}{\text{cos}}^{2}\theta +R_{2}{\text{sin}}^{2}\theta \;$$ (1)

with θ = arctan(*ω*_1_/Ω), where *ω*_1_ is the *B*_1_ field strength (here 1400 Hz) and Ω is the offset from the spinlock carrier frequency. ^1^H-^15^N heteronuclear NOE experiments were recorded on the same samples and consisted of two interleaved experiments, with and without proton saturation, using a recycle delay of 4 s. Spectra were acquired with 64^∗^ x 1024^∗^ complex data points in the indirect (^15^N) and direct (^1^H) dimensions corresponding to acquisition times of 37.6 and 112.6 ms, respectively.

For RNA binding experiments, SERBP1 189–400 or 149–400 samples were diluted to 50 µM in 500 µL in NMR buffer and added to a 5 mm NMR tube. The RNA sequence 5′-GCGCGGG-3′, representing a G-quartet, was synthesized (IDT, IA), desalted, dried, and resuspended in RNAase-free water (Qiagen, MD) to a concentration of 3.125 mM. The RNA stock was titrated into the SERBP1 sample to final RNA:SERBP1 ratios of 2:5, 4:5, 6:5, and 8:5. This required the addition of a maximum of 12 µL of the RNA stock, thus the effect of dilution is negligible. ^1^H,^15^N-HSQC spectra were recorded at 4°C for each titration point with 64^∗^ x 1024^∗^ complex data points in the indirect (^15^N) and direct (^1^H) dimensions corresponding to acquisition times of 37.6 and 112.6 ms, respectively. Spectra were apodized with a sine bell function and zero filled to twice the number of acquired points for data analysis. Chemical shift perturbations (CSP) were calculated by weighting the ^1^H and ^15^N chemical shifts with respect to their gyromagnetic ratio using the following equation (Williamson, 2013):

$$\text{Δδ}=\sqrt{{\left(\text{δ}{\;}^{1}\text{H}\right)}^{2}+0.1{\left({\text{δ}}^{15}\text{N}\right)}^{2}}$$ (2)

CSPs were considered significant when they were higher than the standard deviation of Δδ_max_ for all residues (Williamson, 2013).

### CD Spectroscopy

Circular dichroic spectra were recorded on 10 µM samples of SERBP1 149–400 or 189–400 dissolved in 20 mM Na_2_HPO_4_ pH 6.9, 60 mM NaCl in a 2 mm pathlength circular cuvette using a Jasco 810 spectropolarimeter (Jasco, OK) at a scan speed of 50 nm/ min with 0.5 nm. Temperature was controlled with a recirculating external water bath and was allowed to equilibrate for 20 min before recording data after each ramp. Each temperature point (20–80°C in 10°C steps) was recorded in triplicate, averaged and converted to mean residual ellipticity using previously described relationships (Chemes et al., 2012).

### Size Exclusion Chromatography

Size exclusion chromatography (SEC) experiments were performed using a BioRad NGC fast-performance liquid chromatography system (BioRad, CA, United States) equipped with a Superdex 200 10 x 300 mm analytical size exclusion column (Cytiva, MA, United States), equilibrated with NMR buffer (20 mM Na_2_HPO_4_ pH 6.9, 60 mM NaCl, 1 mM PMSF, 0.2 mM EDTA) in the presence or absence of 2 M guanidinium hydrochloride (GdnHCl). 200 µL of a 15 µM sample of SERBP1 189–400 or 149–400 was applied to the column. The flow rate was 0.5 ml/ min, and elution was monitored at three wavelengths: 215, 280, and 340 nm.

### Analytical Ultracentrifugation

SERBP1 189–400 and 149–400 stock samples were diluted to a concentration of 25 µM into 20 mM Na_2_HPO_4_ pH 7.0, 60 mM NaCl buffer, in presence or absence of 2 M GdnHCl. 360 µL samples were loaded into one sector of a 12 mm double-sector epon-filled centerpiece, and 400 µL of a reference solution of the sample buffer was loaded into the other sector. Radial absorbance scans were collected at 4 min intervals at 280 nm, using an XL-1 analytical centrifuge (Beckman Coulter, CA, United States) equipped with a Ti60 rotor at 40000 rpm, 20°C. Total experiment time was ∼10 h. Data was fit using a continuous c(s) distribution model using the SEDFIT software (Schuck, 2000), using adjusted buffer density (1.04861) and buffer relative viscosity (1.09833) values to account for GdnHCl.

### Dynamic Light Scattering

A SERBP1 189–400 stock sample was diluted to a concentration of 125 µM in 60 µL of 20 mM Na_2_HPO_4_ pH 7.0, 60 mM NaCl buffer, in presence or absence of 2 M GdnHCl, and transferred to a low-volume quartz cuvette. Measurements were conducted on a DynaPro NanoStar dynamic light scattering (DLS) instrument (Wyatt, CA, United States) at 20°C. For each sample 150 10 s acquisitions were collected and averaged. Data were analyzed using the algorithms available in the instrument control software and output as size distributions. Experimentally measured *R*_h_ values were compared with calculated *R*_h_ values using the following equation proposed by Marsh and Forman-Kay to account for intrinsic disorder (Marsh and Forman-Kay, 2010):

$$R_{\text{h}}^{\text{IDP}}=2.49N^{0.509}$$ (3)

where N is the number of residues in the protein polymer chain.

### Phase Separation

SERBP1 149–400 was labeled with the DyLight™ 650 fluorophore (Thermo Fisher Scientific, MO, United States) at the N-terminus using *S. aureus* sortase A following established protocols (Theile et al., 2013; Antos et al., 2017). The sortase-recognition peptide KLPETGG was synthesized, HPLC purified, lyophilized (Genscript, NJ) and reacted with the fluorophore following the manufacturer’s instructions. The fluorescently labeled peptide and SERBP1 were mixed at a 1:1 molar ratio. Recombinant sortase A was added to a final concentration of 2.5 µM and the reaction was allowed to proceed overnight at room temperature. Labeled SERBP1 was separated from unconjugated peptide and free fluorophore by passing over a Superdex 75 HiLoad 16/60 size exclusion column (Cytiva, MA, United States).

Torula yeast RNA (MilliporeSigma, MA, United States) was dissolved at 10 mg/ml in 20 mM Tris pH 7.4, 150 mM NaCl, centrifuged at 13000 g for 10 min, desalted using a PD-10 desalting column (Cytiva, MA, United States), and diluted to a final stock concentration of 3.6 mg/ml determined by A_260_. Unlabeled SERBP1 149–400 was mixed with DyLight 650-labeled SERBP1 149–400 at a molar ratio of 0.03% and dissolved to 6 or 12 µM in 154 mM NaCl, 64 mM Tris pH 7.5, 12.8 % (v/v) glycerol, 1.28 mM DTT, 12.8 % (w/v) PEG3000. Stock RNA was added to a final concentration of 0.4, 0.2, or 0.05 mg/ ml. Samples of SERBP1 189–400 were prepared identically except without fluorescent labeling. Control samples consisting of buffer only or buffer plus RNA only remained clear. SERBP1 samples used in the salt series were prepared similarly except the buffer the protein was dissolved in contained only 20 mM Tris pH 7.5 and 0, 0.05, 0.15, 0.3, or 1 M NaCl.

Phase separation was assayed by transferring 4.5 µL samples to chambered glass coverslips (Grace Biolabs, OR). Chambers were sealed with a second coverslip to reduce evaporation and incubated for 10 min at ambient temperature before imaging with an Olympus FV3000 inverted confocal microscope (Olympus, PA, United States) operating at 1% laser power on the 640 nm channel. Images were acquired simultaneously in differential interference contrast (DIC) and fluorescent modes. Fluorescence recovery after photobleaching (FRAP) measurements were conducted on 12 µM SERBP1 149–400 samples with low (0.05 mg/ ml) or no RNA, and generally recovered to ∼80% of the initial fluorescent intensity. Image contrast was adjusted globally, and droplet area was measured using the appropriate subroutines in Fiji (ImageJ) (Schindelin et al., 2012).

## Results

### SERBP1 is an Atypical RBP Lacking Canonical RNA Binding Motifs

**FIGURE 1:**
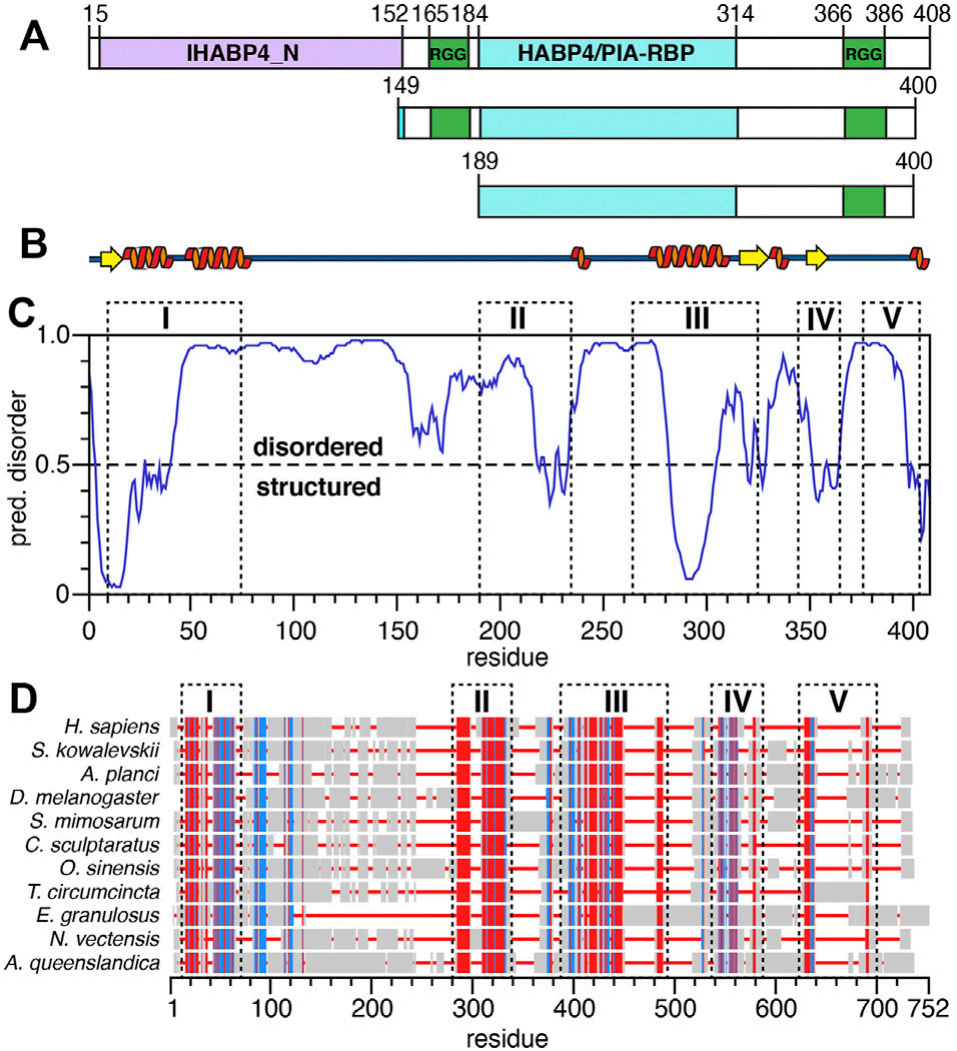
Structural predictions and sequence alignments of human SERBP1. **(A)** Schematic representation of the three SERBP1 constructs. Green boxes represent the RG/RGG repeats, purple and cyan boxes represent the IHABP4 and HABP4 homology domains, respectively. Sequence numbering is from human SERBP1 and indicate the starting and ending amino acids of each domain. **(B)** Representation of SERBP1 secondary structure predicted by PSIPRED. Orange spirals and yellow arrows represent α-helices and β-strands, respectively. **(C)** Disorder plot of SERBP1 predicted by the DISOPRED3 algorithm. Residues with predicted values greater than the 0.5 threshold are predicted as disordered, while residues with values less than the threshold are predicted as structured. **(D)** Sequence alignment of the human SERBP1 sequence with invertebrate homologues. Red denotes conserved residues, blue indicates semi-conserved residues, grey boxes indicate non-conserved residues and red lines indicate gaps. Dashed boxes labeled with roman numerals align the regions of high sequence conservation with predicted regions of order **(panel C)**.

The largest and predominant SERBP1 isoform contains 408 amino-acids encompassing distinct domains including two hyaluronic acid binding protein (HABP) homology domains, IHABP4 (intracellular HABP4, spanning residues 5–152) and HABP4 (residues 189–314) as indicated by UniProtKB entry Q8NC51 (Figure 1A). Two distinct RG/RGG repeats or boxes comprising residues 165–184 and 366–386 are important for RNA-binding and LLPS (Chong et al., 2018). Aside from the RGG boxes, SERBP1 does not contain any other identifiable RNA-binding motif such as an RNA-recognition motif (RRM), zinc-finger, or K-homology (KH) domain (Maris et al., 2005; Valverde et al., 2008; Cook et al., 2015). We used the PSIPRED (Buchan and Jones, 2019) and DISOPRED3 (Ward et al., 2004) webservers to predict regions of secondary structure and intrinsic disorder respectively in SERBP1 (Figures 1B,C). The results of the PSIPRED algorithm, shown as a cartoon representation (Figure 1B), predicts two regions of secondary structure roughly spanning residues 5–80 and 270–360. These results align well with the DISOPRED3 algorithm predictions which indicate SERBP1 is predominantly disordered, except for the first ∼40 N-terminal residues and residues 285–300 (Figure 1C). Several other segments of SERBP1 approach the cutoff threshold for order (e.g., 230–240, 320–330, 350–365, 395–408) and align well with the PSIPRED secondary structure predictions indicating the potential for transiently-formed structure.

SERBP1 is highly conserved among vertebrates, thus to gain insight into the functional relevance of the different domains of SERBP1, we aligned human SERBP1 to several homologous proteins from invertebrates (Figure 1D). The alignments are shown with gaps, and dashed boxes denote regions with conserved (red) or semi-conserved (blue) sequence homology. Full alignments are presented in Supplementary Figure S1. Notably, the highly homologous regions align well with the predicted secondary structure (Figure 1B) and ordered regions (Figure 1C), in particular residues 285–300, which are predicted to be helical, are highly conserved (region “III” in Figures 1C,D), indicating a potentially crucial role for SERBP1 function. Moreover, it appears that although the two RGG boxes show high conservation among vertebrates, these regions are highly heterogeneous among non-vertebrates (Figure 1D; Supplementary Figure S1). To expand on the role of the RGG boxes for SERBP1 function, we designed two truncated constructs: 149–400 or 189–400 which contain either both (149–400) or only the C-terminal (189–400) RGG box(es) (Figure 1A). In both constructs, we also removed eight C-terminal residues (401–408) that are hydrophobic and contributed to instability and were consistently degraded from SERBP1. To confirm that the truncated proteins faithfully reproduce the structural features of the full-length protein, ^1^H,^15^N-HSQC spectra were recorded. Overlays of the spectra from full-length SERBP1 with spectra from each of the truncation proteins are almost identical indicating that the truncations did not lead to major structural changes (Supplementary Figure S2). The N-terminal region, that was predicted as structured, was not detected as such in the full-length spectrum, and thus we decided to focus our study on the truncated proteins.

### Structural Characterization of SERBP1 Indicates the Presence of a Stable α-Helix

**FIGURE 2:**
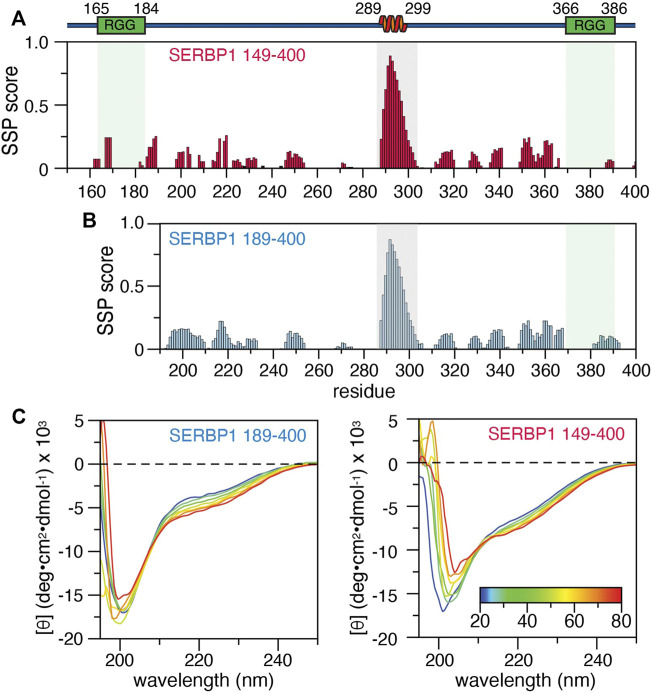
Secondary structure of SERBP1 149–400 and 189–400. Secondary structure propensity calculated from backbone chemical shifts by the SSP algorithm for **(A)** SERBP1 149–400 and **(B)** 189–400. Gray boxes outline residues 289–299, identified as having a high α-helical character. A cartoon representation of the α-helix and relative position of the RGG boxes (green) is shown above the plots. **(C)** CD spectra of SERBP1 189–400 **(right)** and SERBP1 149–400 **(left)** acquired at 10°C intervals from 20°C to 80°C.

We previously reported the backbone resonance assignments for SERBP1 189–400 (BMRB accession number 50953) and here we report the backbone resonance assignments for SERBP1 149–400 (Supplementary Figure S3). Over 77% of the backbone resonances from the 252 residue SERBP1 149–400 were assigned, excluding the 11 proline residues. Of the 56 residues not assigned, 32 are arginines or glycines that belong to the two RG/RGG repeats and thus, because of the inherent sequence degeneracy in these regions, could not be unambiguously assigned. The remining 24 residues were either ambiguous or were severely overlapped in the spectrum. Overall, assignments of SERBP1 149–400 Cα, Cβ, and C′ chemical shifts were 81, 84.6, and 77.8% complete respectively. Analysis of the Cα, Cβ, C’, H_N_, N, and Hα chemical shifts using the Secondary Structure Propensity (SSP) algorithm (Marsh et al., 2006) indicate that the majority of SERBP1 189–400 is disordered, except for residues 289–299, which have significant α-helical character (Figure 2A). A similar analysis for the 149–400 construct revealed α-helical propensity for the same residues (Figure 2B), indicating that the first RGG domain does not influence the formation or stability of the helix. Similar results were obtained with the δ2D (Camilloni et al., 2012) and TALOS-N (Shen and Bax, 2013) algorithms which indicate substantial (>80 %) α-helical propensity for residues 289–299 in both the SERBP1 149–400 and 189–400 constructs (Supplementary Figure S4). Circular dichroic (CD) spectra of SERBP1 149–400 and 189–400 recorded to measure thermal denaturation have a similar appearance and contain features indicative of IDPs such as the strong negative transition at 200 nm (Figure 2C). For both constructs, a negative transition at approximately 222 nm becomes more negative as the temperature is increased from 20 to 80°C. The temperature gradient also reveals an isodichroic point at 212 nm indicative of a two-state transition that could translate to exchange between partially ordered and completely disordered conformations (Greenfield, 2006). Deconvolution of these CD spectra to estimate helical content is complicated by the inherent contributions from polyproline II conformations that contribute to the observed transitions at 200 and 212 nm (Kjaergaard and Poulsen, 2011). Taken together these data are consistent with the chemical shift analysis (Figures 2A,B), secondary structure, and disorder predictions Figures 1B,C and indicate that SERBP1 is primarily disordered with some α-helical character.

**FIGURE 3:**
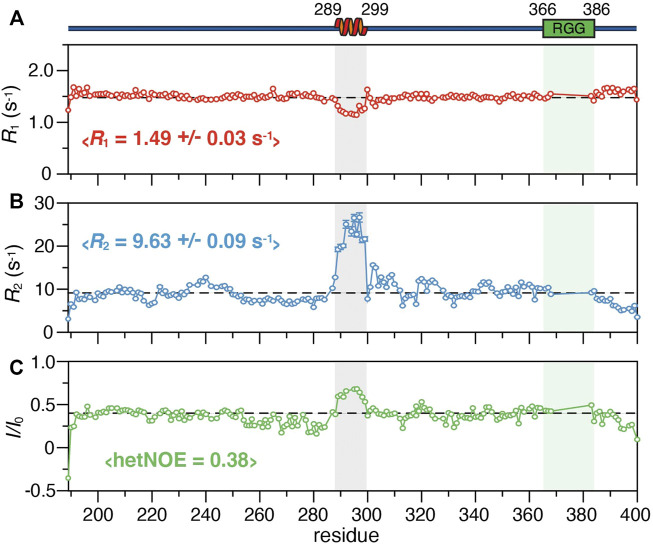
^15^N relaxation parameters of SERBP1 189–400 including **(A)** *R*_1_ and **(B)** *R*_2_ rates, and **(C)** the ^1^H-^15^N heteronuclear NOE plotted against the protein sequence. The dashed lines represent the average value for each experiment, also indicated in brackets. A cartoon representation of the α-helix identified from chemical shift information and relative position of the RGG boxes (green) is shown above the plots. The shaded boxes align the helical region and RGG box over all panels.

To further define the structural properties of SERBP1, we investigated the fast timescale dynamics of the truncation mutants by measuring the ^15^N *R*_1_ and *R*_2_ relaxation rates, as well as the ^1^H-^15^N heteronuclear NOE (Figure 3; Supplementary Figure S5). For the 189–400 construct, the average *R*_1_ and *R*_2_ values are 1.5 s^−1^ and 9.6 s^−1^, respectively, except for residues 289–299 in the region identified as helical by SSP, which have average *R*_1_ and *R*_2_ of 1.2 s^−1^ and 22.5 s^−1^ respectively. We observed similar average *R*_1_ and *R*_2_ (1.5 s^−1^ and 9.5 s^−1^ respectively) along with variations in the same region (289–299) for SERBP1 149–400 (Supplementary Figure S5). Average heteronuclear NOE values for both constructs were 0.4 with the residues in the 289–299 stretch displaying values approaching 0.75, indicating the motions of these amino acids are more restricted than the rest of the chain. These data reveal that the two truncated proteins retain virtually identical dynamics and contain a stable α-helix comprising residues 289–299, the stability of which seems to be independent of the RGG boxes.

### SERBP1 Behaves as a Compact, Monomeric IDP

**FIGURE 4:**
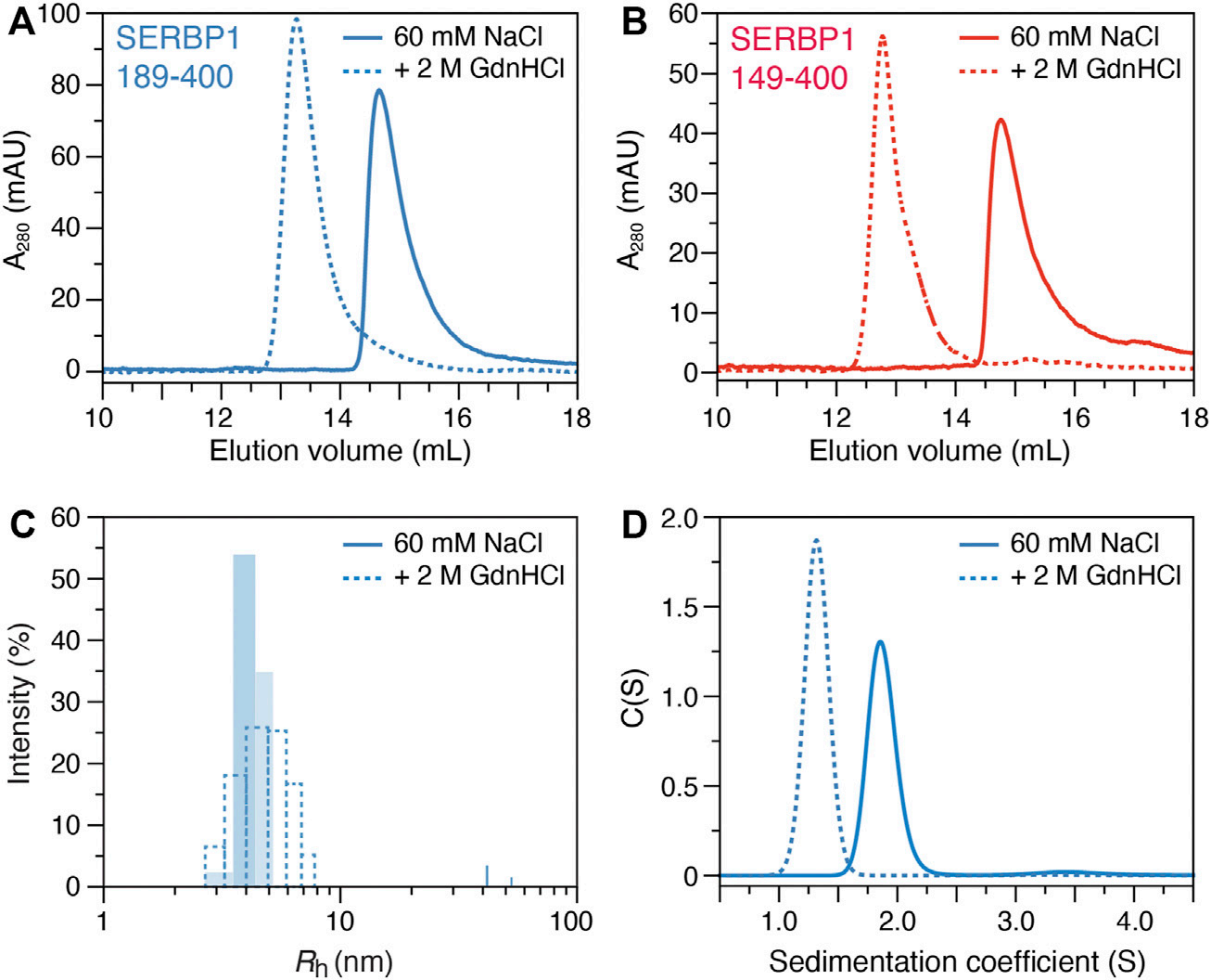
Biophysical characterization of SERBP1 oligomer state in solution. Size exclusion chromatography profiles of **(A)** SERBP1 189–400 and **(B)** SERBP1 149–400, respectively, in the absence (solid) and presence (red) of 2 M GdnHCl recorded at A_280_. **(C)** Size distribution histograms of the apparent hydrodynamic radius (*R*_h_) derived from dynamic light scattering experiments of SERBP1 189–400 in the absence (solid) and presence (dashed) of 2 M GdnHCl. **(D)** Analytical ultracentrifugation sedimentation velocity coefficient profiles of SERBP1 189–400 in the absence (solid) and presence (dashed) of 2 M GdnHCl.

Size exclusion chromatography (SEC), dynamic light scattering (DLS) and analytical ultracentrifugation (AUC) were employed to assess the oligomeric state of SERBP1 and to determine if the stable α-helix acts as a dimerization interface, as has previously been described for TAR DNA-binding protein (TDP-43) (Conicella et al., 2016). At physiological conditions, SERBP1 189–400 elutes at 14.7 ml (Figure 4A), which, according to column calibration (data not shown) corresponds to a molecular weight of 51.6 kDa, suggesting a possible dimerization of SERBP1 (the molecular weight of monomers is 23.7 kDa) if SERBP1 were a globular, folded protein. However, due to their extended conformations, IDPs are expected to elute earlier in SEC corresponding to larger apparent molecular weights (Uversky, 2012). The SEC experiment was repeated in the presence of 2 M of GdnHCl, resulting in an elution volume of 13.3 ml, corresponding to a molecular weight of 95.9 kDa (Figure 4A). Similar behavior was observed for SERBP1 149–400 which displayed elution volumes of 14.8 and 12.8 ml in absence and in presence of 2 M GdnHCl, respectively (Figure 4B). If SERBP1 formed oligomers in the absence of GdnHCl, the elution volume should increase upon the addition GdnHCl, contrary to the observed decrease. These results suggest that SERBP1 becomes more extended, occupying a much larger steric volume in 2 M GdnHCl than in physiological conditions.

To further assess the oligomeric state of SERBP1, DLS was used to measure the apparent hydrodynamic radius (*R*_h_) at physiological conditions and in the presence of 2 M GdnHCl (Figure 4C). A mean *R*_h_ estimation of 3.9 and 4.4 nm under physiological and denaturing conditions, respectively, was obtained for SERBP1 189–400. Notably, the polydispersity in the presence of GdnHCl was ∼30%, higher than the ∼13% that was observed under non-denaturing conditions. The small relative difference in *R*_h_ in the presence and absence of GdnHCl indicates that SERBP1 is a monomer at physiological conditions, consistent with the SEC results, adopting a conformation that is more compact than a fully disordered chain. The theoretical *R*_h_ value, calculated by the method proposed by Marsh and Forman-Kay (Marsh and Forman-Kay, 2010) was 3.8 nm, in very good agreement with the experimentally measured value of 3.9 nm, further evidence that SERBP1 is monomeric at these conditions (Figure 4C). Finally, AUC experiments were conducted on SERBP1 189–400 in absence and presence of 2 M GdnHCl (Figure 4D). The fitted sedimentation coefficient was lower (1.3 *vs.* 1.9) and the frictional ratio was higher (2.2 *vs.* 1.8, not shown) in presence of GdnHCl, indicating SERBP1 189–400 is more compact under physiological conditions than a fully extended polypeptide chain, and that it becomes less compact in denaturing conditions. Similar results were obtained for SERBP1 149–400 with a lower sedimentation coefficient (1.5 *vs.* 2.2) and a higher frictional ratio (2.1 *vs.* 1.7, not shown) in the presence vs. absence of 2 M GdnHCl respectively (Supplementary Figure S6). These observations are consistent with the average *R*_2_ rates recorded for both constructs, (∼9.5 s^−1^) which are faster than expected for proteins that would preferentially exist in extended conformations (Figure 3B; Supplementary Figure S5B). Together, these data are highly consistent and reveal that SERBP1 is monomeric under the experimental conditions used here, adopting conformations that are more compact than a fully extended polymer.

### SERBP1 Interacts With Guanine-Rich RNA

**FIGURE 5:**
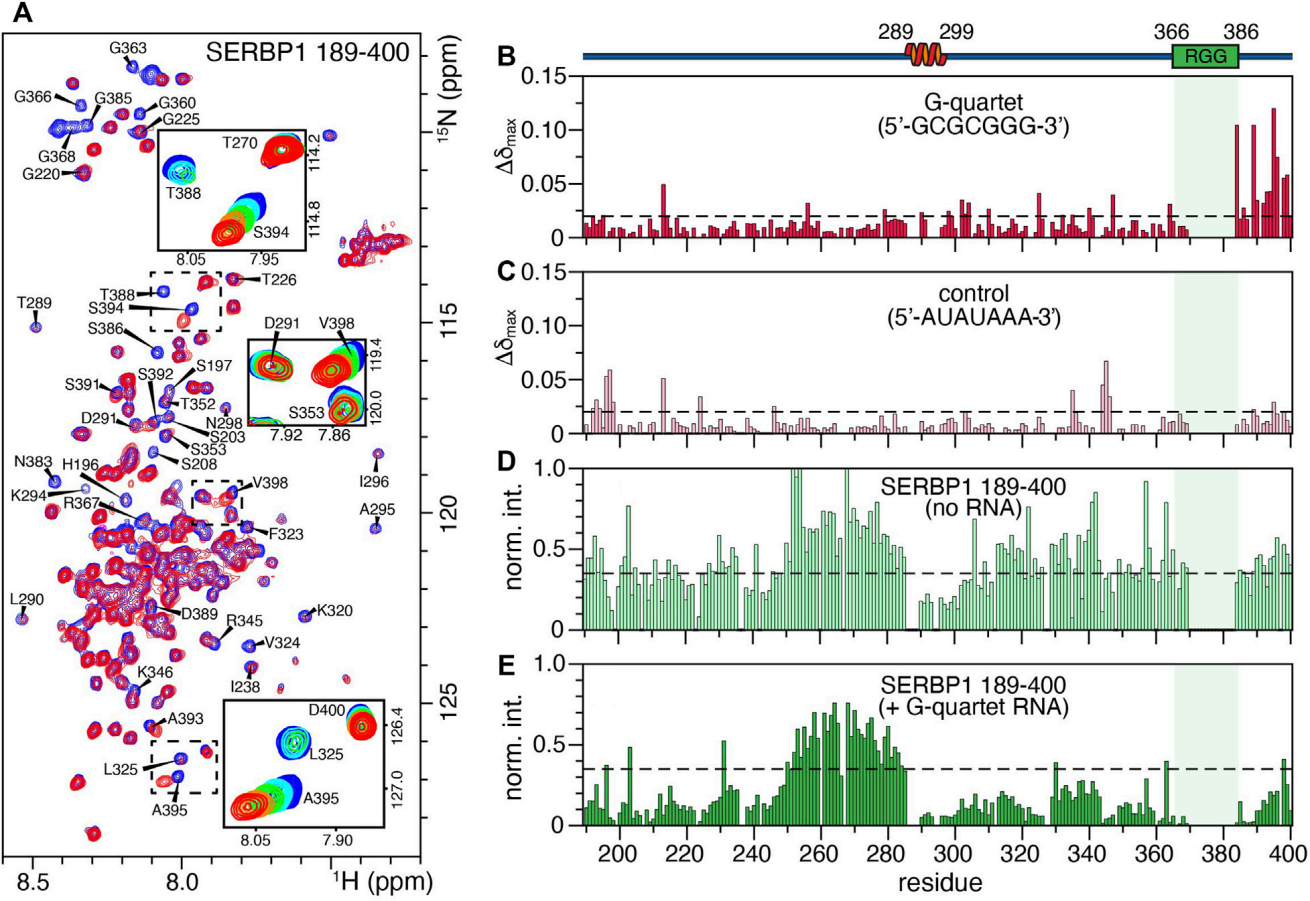
Binding of SERBP1 189–400 to a 7-mer RNA oligonucleotide measured by chemical shift perturbations (CSP). **(A)** Overlay of the ^1^H,^15^N-HSQC spectra of SERBP1 in absence (blue) and presence (red) of 1:1.6 molar ratio of the 5′-GCGCGGG-3′ RNA 7-mer. Only the two titration points are shown to highlight the broadening of glycine residues observed upon addition of RNA ligand. The insets show the full titration series of peaks that shift significantly. A cartoon representation of the α-helix identified from chemical shift information and relative position of the RGG boxes (green) is shown above the plots. The shaded box aligns the RGG box over all panels. **(B)** Maximum CSP (Δδ_max_) induced by the RNA G-quartet ligand binding plotted against SERBP1 189–400 sequence. The dashed line represents the mean standard deviation of Δδ_max_ for all peaks, shifts above this threshold are considered significant. **(C)** Maximum CSP (Δδ_max_) of a negative control RNA ligand (5′-AUAUAAA-3′) plotted against SERBP1 189–400 sequence. The dashed line represents the mean standard deviation of Δδ_max_ for all peaks. Normalized intensities of the ^1^H,^15^N-HSQC peaks of SERBP1 189–400 plotted as a function of the protein sequence, in **(D)** the absence and **(E)** presence of 1:1.6 molar ratio of protein to the RNA 7-mer G-quartet ligand. Dashed lines are the same in both panels and represent the average intensity of SERBP1 peaks in the absence of RNA.

SERBP1 is thought to be an important regulator of many different mRNAs although the structural details of RNA recognition remain unclear. Previously, the RNAcompete assay defined 5′-GCGCGGG-3′ as the SERBP1 consensus binding motif and subsequently it was shown that an RNA oligonucleotide containing this sequence binds full-length SERBP1 with sub-micromolar affinity (Kosti et al., 2020). Here, heteronuclear NMR was used to study the interaction between SERBP1 189–400 and the 7-mer RNA oligonucleotide. A series of ^1^H,^15^N heteronuclear single quantum correlation (HSQC) spectra of SERBP1 189–400 were acquired in the presence of increasing concentrations of the 7-mer RNA (Figure 5A). Overall, the spectra remain similar in response to increasing concentrations of the 7-mer indicating the RNA ligand does not induce folding of SERBP1, a phenomenon that has been observed for other IDPs (Sugase et al., 2007; Tompa and Fuxreiter, 2008; Shammas et al., 2016; Bonetti et al., 2018; Robustelli et al., 2020). However, some peaks display strong chemical shift perturbations (CSP), such as A395, S394, and V398, indicating these residues interact strongly with the RNA (Figure 5A, insets). Additionally, glycine peaks (e.g., G363, G366) belonging to the RGG box broaden significantly as the ligand concentration increases, likely due to exchange between the free and the bound forms at an intermediate rate on the NMR time scale. These observations lead to the proposal that electrostatic interactions occur between the positively charged arginine residues of the RGG box and the negatively charged phosphates of the RNA backbone. This encounter complex could then be stabilized by interactions with residues N383-V398, especially with T388 and S394 since serine and threonine are excellent hydrogen bond donors. Plotting the maximal CSP (Δδ_max_) against the protein sequence reveals that the RNA interacts with residues spanning from the second RGG box to the C-terminus of SERBP1, with some Δδ_max_ higher than 0.1 ppm (e.g., N383, T388, S394) (Figure 5B). If an initial SERBP1:RNA encounter complex forms through charge-charge interactions, the complementary RNA sequence (5′-AUAUAAA-3′) may induce similar broadening in the RGG-specific glycine residues. No specific CSPs were observed in the C-terminal region of the SERBP1 189–400 (Figure 5C), and broadening of resonances belonging to the RGG box was not detected (Supplementary Figure S7). Some CSPs higher than 0.05 ppm are randomly dispersed along the chain, but these are more likely indicative of non-specific protein-RNA interactions. Taken together, these data reveal that further experimentation is required to fully elucidate the nature of the SERBP1 RNA recognition and complex formation.

The relative peak intensities of SERBP1 residues before and after titration were plotted as a function of the amino acid sequence to gain insight into the global effect of the RNA binding on the protein. (Figures 5D,E). Uniform broadening of the peaks along the entire protein sequence was observed upon the addition of stoichiometric quantities of the 7-mer RNA, with the exception of residues 250–285, that precede the α-helix. Notably, these residues retain approximately equal intensities in the absence or presence of the RNA (compare Figures 5D,E), suggesting they remain unaffected by interactions with RNA. This region has lower than average heteronuclear NOE (∼0.25) and *R*_2_ rates (∼7 s^−1^) indicating that these residues are more dynamic compared to the rest of the protein and remain mobile in the presence of RNA (Figures 3B,C). Finally, no CSPs or peak broadening beyond the uniform broadening described above were observed for residues 289–299 that comprise the α-helix indicating that this region is not involved in recognition or binding of the RNA 7-mer.

### SERBP1 Phase Separation is Inhibited by the Presence of Salt and Modulated by RNA

Intrinsically disordered RBPs are overrepresented in the group of proteins that are known to participate in biomolecular condensates and undergo LLPS (Burke et al., 2015; Elbaum-Garfinkle et al., 2015; Boeynaems et al., 2017). The phase separation properties of the SERBP1 149–400 and 189–400 constructs were assayed using droplet formation assays and fluorescence recovery after photobleaching (FRAP). At neutral pH, SERBP1 149–400 readily phase separates at a concentration of 10 µM in absence of NaCl. The addition of NaCl abrogates SERBP1 phase separation with noticeably smaller and fewer droplets forming in the presence of 50 mM NaCl and complete dissipation at 150 mM NaCl (Supplementary Figure S8A). Conversely, SERBP1 189–400 does not phase separate at all under the same physiochemical conditions (Supplementary Figure S8B). Increasing NaCl concentrations (50–1000 mM) had no effect on the LLPS characteristics of SERBP1 189–400. Since the main difference between the two constructs is the N-terminal RGG box, it is reasonable to conclude that the RGG boxes promote phase separation of SERBP1, consistent with what has been described for other RGG-containing RBPs (Chong et al., 2018).

**FIGURE 6:**
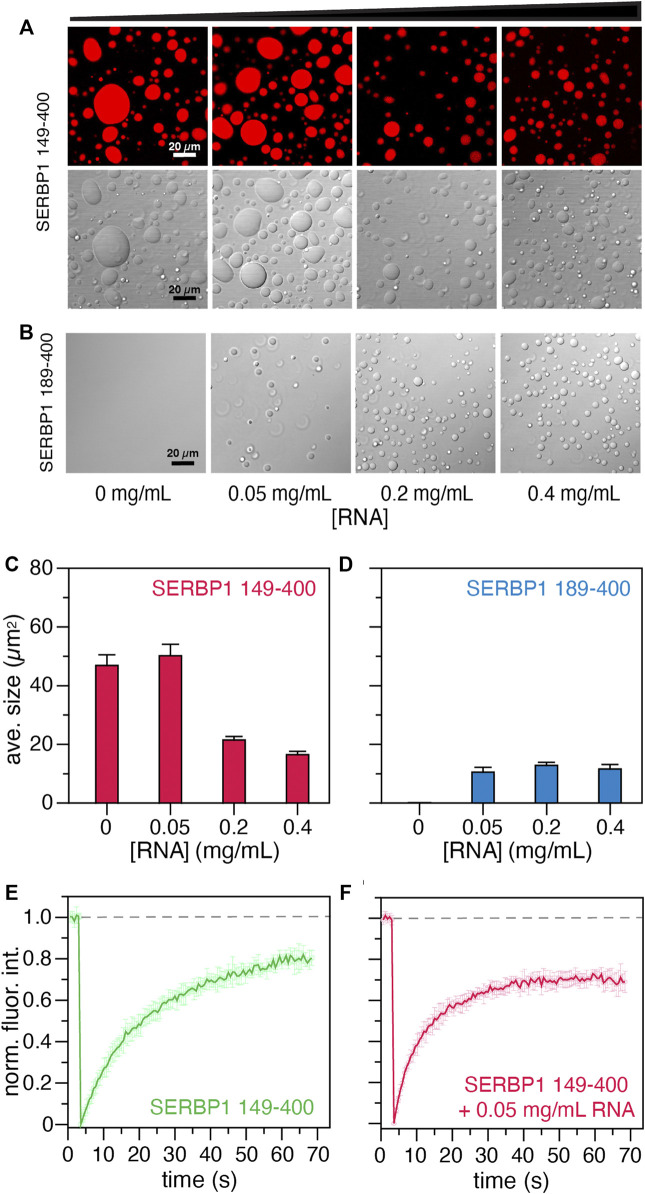
Phase separation assays of SERBP1 149–400 and SERBP1 189–400. Effect of RNA on the LLPS propensity of 12 µM **(A)** SERBP1 149–400 or **(B)** SERBP1 189–400 in buffer containing 13 % (v/v) glycerol and 13 % (w/v) PEG3000. Torula yeast RNA was titrated into SERBP1 protein solutions to final concentrations ranging from 0 to 0.4 mg/ ml. The average droplet sizes (µm^2^) shown for **(C)** SERBP1 149–400 and **(D)** SERBP1 189–400 were derived from three independent images. Normalized fluorescence recovery after photobleaching (FRAP) profiles of 12 µM SERBP1 149–400 in the **(E)** absence or **(F)** presence of 0.05 mg/ ml torula yeast RNA. Dashed line indicates average the fluorescence before photobleaching.

Given the observed effects of NaCl on phase separation of SERBP1, the effect of RNA was also tested. In this assay, 150 mM NaCl, 13 % glycerol, and 13% PEG3000 were included in the droplet formation buffer with 12 µM SERBP1 149–400 or SERBP1 189–400 and increasing concentrations of torula yeast RNA (Figures 6A,B). Contrary to the effect of NaCl, RNA promoted phase separation of SERBP1 189–400 at concentrations as low as 0.05 mg/ ml RNA (Figure 6B). Under the three RNA concentrations tested (0.05, 0.2, and 0.4 mg/ ml), the observed average droplet size for SERBP1 189–400 remained consistent at ∼12 μm^2^ (Figure 6D). At the same protein concentration, phase separation of SERBP1 149–400 was promoted by the presence of glycerol and PEG3000 (compare the 0 mg/ ml panels in Figures 6 A,B) while droplet size strongly correlated to the RNA concentration (Figure 6A). At no added RNA or 0.05 mg/ ml RNA, the average observed droplet size for SERBP1 149–400 was ∼50 μm^2^. The average droplet size significantly decreased to ∼20 μm^2^ as the RNA concentration increased to 0.2 and 0.4 mg/ ml (Figure 6C). This trend held when the experiment was repeated with 6 µM SERBP1 149–400 albeit the average observed droplet size was lower, ranging from 10 μm^2^ without RNA to 2 μm^2^ at an RNA concentration of 0.4 mg/ ml (Supplementary Figure S9). RNA modulates SERBP1 phase separation, promoting phase separation for SERBP1 189–400 under non-permissive conditions, as well as altering the droplet characteristics formed from SERBP1 149–400. While the influence of crowding agents cannot be separated from the contribution of the RGG boxes to phase separation in these experiments, taken together these results show that the RGG boxes are important for SERBP1 LLPS, which likely proceeds through a charge-mediated mechanism.

The liquid -like nature of the condensates formed by SERBP1 149–400 in the presence and absence of RNA were probed using FRAP experiments (Figure 6D). In the absence of RNA, the recovery of fluorescence plateaus at ∼70 s, while in the presence of 0.05 mg/ ml of RNA, the fluorescence intensity recovers faster, reaching a plateau in ∼40 s. The faster recovery of fluorescence in the presence of RNA is indicative of faster diffusion and hence more dynamic droplets. These results support the droplet formation assays suggesting that the two RGG boxes might interact synergistically with the RNA to modulate the condensate behavior of SERBP1. Further investigation will be necessary to decipher the molecular mechanisms of SERBP1 LLPS.

## Discussion

SERBP1 was initially identified as a hyaluronic acid binding protein in biochemical pull-down studies using hyaluronic acid as bait (Heaton et al., 2001). The pattern of expression in normal tissue of the two known human hyaluronic acid binding proteins, HABP2 and HABP4 are different than SERBP1. HABP2 is predominantly found in the liver, while expression of HABP4 is the opposite of what was found for SERBP1 with high expression in all regions of the brain and very low expression in immortalized cells (Kosti et al., 2020). HABP4 binds mRNA and is known to interact with Receptor of activated protein C kinase (RACK1), a protein implicated in mRNA splicing and translation, although the significance of these associations is unknown (Huang et al., 2000; Nery et al., 2004). Similarly, SERBP1 was recently identified as a binding partner of RACK1 (Bolger, 2017), an interaction that was proposed to play a role in ribosomal composition and translation regulation. Thus, it is possible that although SERBP1 and HABP4 share significant sequence homology, the similarities are coincidental and represent an example of convergent or parallel evolution (Storz, 2016).

Sequence alignments with non-vertebrate SERBP1 homologues revealed five broadly defined regions of homology that roughly correlate with predicted SERBP1 ordered structure (compare dashed boxes in Figures 1C,D). In particular, chemical shift-derived secondary structure propensity, and CD measurements support predictions that SERBP1 is not entirely disordered but rather contains stable native structure in the form of an α-helix (residues 289–299), a structural feature common to many IDPs (Lee et al., 2012b), in a stretch of residues that are highly conserved among vertebrate and invertebrate sequences. The α-helix appears stable in both the 189–400 and 149–400 constructs and displays faster than expected transverse (*R*_2_) relaxation rates on the order of 20–25 s^−1^. These observations suggested the α-helix might be stabilized by intra- or intermolecular associations, or possibly mediate homodimerization. Such behavior has been described for TDP-43, a protein involved amyotrophic lateral sclerosis, whose disordered C-terminus domain contains an α-helix involved in protein-protein homodimerization and liquid-liquid phase separation (Conicella et al., 2016; Lim et al., 2016; Conicella et al., 2020). Therefore, intramolecular interactions of SERBP1 may contribute to the stability of the helix and promote preferential sampling of compact conformations, as observed for other IDPs (Marsh and Forman-Kay, 2010). Alternatively, the α-helix, or the other conserved domains might act as molecular recognition motifs, mediating associations with various biomolecular partners, through folding-upon-binding mechanisms (Sugase et al., 2007). Relaxation dispersion experiments did not reveal exchange with alternate SERBP1 conformers, yet these experiments do not rule out this possibility since the exchange timescale may be inaccessible for CPMG-based experiments. Paramagnetic relaxation enhancements, filtered NOE, or off-resonance *R*_1ρ_ experiments maybe more suitable to probe SERBP1 conformational exchange and are an active area of investigation.

SERBP1 is a multifunctional mRNA binding protein that has roles in regulating the expression of several mRNAs yet despite clear evidence of RNA binding (Heaton et al., 2001; Anger et al., 2013; Muto et al., 2018) it does not seem to possess any canonical RNA binding motifs, such as RRMs, zinc-fingers or KH domains, which are predominant in other RNA-binding proteins (Maris et al., 2005; Valverde et al., 2008; Cook et al., 2015). The recognizable RNA-binding features of SERBP1 are two RG/RGG repeat regions comprising residues 165–184 and 366–386. CSPs and differential peak broadening suggest that the G-quartet RNA interaction spans the C-terminal RGG box and incorporates adjacent residues. Mutagenesis experiments will be useful to further define the role of the RGG box as well as uncover the source of the specificity of guanines over the other nucleotides (Figures 5B,C). Furthermore, these results are consistent with recent observations that some RBPs, particularly intrinsically disordered RBPs, can interact with RNA without the presence of specific folded RNA-binding domains (Castello et al., 2016; Järvelin et al., 2016; Hentze et al., 2018). As described here for SERBP1, RGG repeats of this class of RBP seem to play an important role in mediating interactions with RNA (Chong et al., 2018). For example, the RGG box of fragile X mental retardation protein (FMRP) was shown to interact with G-quadruplex forming RNA sequences (Darnell et al., 2001; Phan et al., 2011). Since SERBP1 was recently shown to bind G-quadruplexes (Su et al., 2021), it is important to elucidate the binding mode, the relative contribution of both RGG boxes, and any involvement of other conserved sequence motifs. A particularly important question to investigate is if SERBP1 actively stabilizes or destabilizes G-quadruplexes.

Proximity labeling approaches have identified SERBP1 interactions with myriad RBPs involved in mRNA regulation, stabilization, and splicing, translation, neurogenesis, synaptogenesis, and ribosome binding such as CAPRIN1, EIF4B, FXR1, LARP1, PABPC4, SYNCRIP, and YTHDF3 among others (Youn et al., 2018; Pedley et al., 2020). Similar to SERBP1, the subcellular localization of many of these proteins is controlled by arginine methylation, enabling them to shuttle between the nucleus and cytoplasm (Lee et al., 2012a). Indeed, many of these proteins localize to stress granules or other subcellular biomolecular condensates (Lee et al., 2014), and likewise, SERBP1 was shown here to undergo LLPS mediated by its RGG boxes. SERPB1 149–400 readily phase separates in low ionic strength buffers at neutral pH while the 189–400 construct does not, highlighting the importance of the presence of both RGG boxes to promote LLPS (Figure 6). The response to increasing ionic strength indicate that electrostatic interactions are an important mechanism mediating the transient self-associative contacts responsible for condensate formation as has been described for the BRD4-IDR (Sabari et al., 2018; Han et al., 2020) or Ddx4 (Brady et al., 2017). Indeed, LLPS of protein-RNA complexes has recently been described as a way of regulating biological processes such as transcriptional or translational events (Han et al., 2012; Berry et al., 2015; Smith et al., 2016), and in some cases, RNA has been proposed to slow the formation of droplets of aggregation-prone prion-like proteins (Maharana et al., 2018). While RNA was shown to impact the LLPS properties of SERBP1, the effect of specific RNA structures like stem-loops or G-quadruplexes on SERBP1 condensate formation will require further investigation. Additionally, the α-helix in SERBP1 may contribute to electrostatic contacts important for LLPS since several residues comprising the α-helix are charged (E287, D291, E292, K294, D300).

Genomic studies indicate SERBP1 has a significant role in the one-carbon metabolism cycle and was implicated in synaptogenesis and neuronal development (Kosti et al., 2020). These pathways seem to be important in the development and progression of GBM. Indeed, SERBP1 is overexpressed in the brains of GBM patients and is negatively correlated with a favorable prognosis (Kunkle et al., 2013). Involvement of SERBP1 in these pathways may be through regulation of mRNA translation, transcription or splicing or through a combination of these functions. For example, SERBP1 was identified bound to the mRNA tunnel in the structures of inactive 80 S ribosomes reported by different groups (Anger et al., 2013; Brown et al., 2018; Muto et al., 2018), and was hypothesized to aid in translational control by negatively regulating ribosomal activity. The significance of these discoveries is not fully understood but may be related to a feedback cycle linking mRNA splicing to transcription mediated by RBPs like SERBP1. Recent work revealed a similar function for the SARS-CoV-2 protein Nsp1, which inserts its C-terminal domain into the ribosome mRNA channel, interrupting host transcriptional regulation (Schubert et al., 2020). Further, Zhang et al., found that SARS-CoV-2 also dysregulates One-carbon metabolism by increasing *de novo* purine synthesis and glycolysis (Zhang et al., 2021), a mechanism that was recently described for SERBP1 in GBM metabolism regulation (Kosti et al., 2020). These findings require further investigation to determine whether this is a specific strategy involving host mimicry of SERBP1 or simply a consequence of the general viral infection strategy of disrupting host translational regulation.

In summary, SERBP1 is an atypical RBP lacking known RNA recognition motifs and has multifunctional roles in translation and mRNA regulation and modulates One-carbon metabolism, neuronal differentiation and synaptogenesis. We identified the G-quartet RNA binding site on SERBP1 using NMR and present several lines of evidence such as conserved secondary structure and semi-compact conformations that suggest SERBP1 also recognizes higher-order RNA structures including stem-loops and G-quadraplexes. Current efforts are focused on identifying SERBP1 interactions with G-quadruplexes and assaying for folding or destabilizing activity. Additionally, the partially folded and compact nature of SERBP1 may be indicative of pre-encounter conformations important for recognition and binding to other biomolecular targets. Future studies will examine the conformational dynamics of the α-helix and neighboring regions as potential binding sites for the myriad identified binding partners.

## Data Availability

The raw data supporting the conclusions of this article will be made available by the authors, without undue reservation. The expression plasmid encoding SERBP1 149-400 was deposited with Addgene (176516) and the backbone resonance assignments are available from the Biological Magnetic Resonance Bank (51080).
