# Supplementary material for: Structural Characterization of the RNA-Binding Protein SERBP1 Reveals Intrinsic Disorder and Atypical RNA Binding Modes
Source: Front Mol Biosci. 2021 Sep 24;8:744707. doi: 10.3389/fmolb.2021.744707 (PMC8497785; doi:10.3389/fmolb.2021.744707)
Supplement: Supplementary file 1 [file DataSheet1.docx]

**Supplementary Material**

**Structural characterization of the RNA-binding protein SERBP1 reveals intrinsic disorder and atypical RNA binding modes.**

**Antoine Baudin^1,2^, Alma K. Moreno-Romero^1,2^, Xiaoping Xu^1,2^, Emily E. Selig^1,2^, Luiz O. F. Penalva^1,3^, David S. Libich^1,2^***

^1^Greehey Children’s Cancer Research Institute, The University of Texas Health Science Center at San Antonio, San Antonio TX, United States

^2^Department of Biochemistry and Structural Biology, The University of Texas Health Science Center at San Antonio, San Antonio TX, United States

^3^Department of Cell Systems and Anatomy, The University of Texas Health Science Center at San Antonio, San Antonio TX, United States

**Supplementary Table 1:** Primers used in the construction of SERBP1 149-400, 189-400, and 1-408 (full-length) expression constructs.

| Construct | Primer Name | Sequence |
| --- | --- | --- |
| SerBP1-149-400 | SERBP1-149 Forward* | *TTTCAGGGCGCC*GGCGAATTCAGCGTTGACCG |
|  | SERBP1-400 Reverse** | *CGCGGATCCTTA*ATCATCAACATCCGGAGCGC |
| SerBP1-189-400 | SERBP1-189 Forward | *TTTCAGGGCGCC*GGTAAGCGTGAGTTCGACCG |
|  | SERBP1-400 Reverse | C*GCGGATCCTTA*ATCATCAACATCCGGAGCGC |
| SerBP1-1-408 | SERBP1 Forward | *TTTCAGGGCGCC*ATGCCGGGTCACCTGCAGG |
|  | SERBP1-408 Reverse | *CGCGGATCCTTA*CGCCAGAGCCGGGAAAG |

* All forward primers have *Kas*I sites in italic

** All reverse primers have *Bam*H1 sites and stop codon in italic.

***H. sapiens*** 1 mpghLQE-------gFGCVVTNRFDQLF--**D**DE-S**DP**-----FEVLKAAENKKKEAGGGGVGGPG--------AKSAaQA 57

***S. kowalevskii*** 1 ----MDC-S------YGIGVANRYALFL--**D**SE-V**DP**dalndVEDQKKDQEKPSKKQDKRAKGEA-------KSKQN-QQ 58

***A. planci*** 1 ----MDS-Q------YSVQVKNRFYLDM--**D**MvG-**DP**-----LEILPTETKSQKKDKKKKSEGKK--------------- 46

***D. melanogaster*** 1 ---------------MDSAGKNRYELLF-m**D**DDvS**DP**-----LDNLVAPTAAAAAAAAGKKKQPS----------AAaAA 49

***S. mimosarum***  1 ----MEN-A------YGIGVKNRYELFY--**D**ED-V**DP**-----LEIIRQQEEEKEKRKTDKHCKD--------KTKVS-KS 52

***C. sculpturatus*** 1 ----MET-T------YGVGIKNRYELFY--**D**DE-E**DP**-----LEILRQQQKETEKKKVEKVPKNA-------KTKGT-KP 53

***O. sinensis*** 1 ----MES-Q------YGIVVSNKYSLFY--**D**D--A**DP**-----LDMLHQQEAKVKKKDTVKKEVD--------KSKAN-KA 51

***T. circumcincta*** 1 ------MvE------YGCNVANKFGFMS-d**D**DEfD**DP**qelisRVTQLEAEKAAAQKKADKLAKQA---avapKEPVK-AA 63

**E. granulosus** 1 ---m---tSavhdytYSVQVKSRFDLFAkd**D**YNgE**DP**dvl--LTRLRTQKRERKSSAKEASEKASqdvl-epIQKET--Q 69

***N. vectensis*** 1 ----MDAvE------YSIGVNNRFGLLL-s**D**EE--**DP**------ETTFKESEKAAKETKDKKAKSA--------------- 46

***A. queenslandica*** 1 ----MET-A------FSVATNNRFAFFM--**D**DE-D**DP**-----GDVTGGQSSQEKKGKAEGGKQPQqptggspKNKENgQT 61

**I**

***H. sapiens*** 58 AAQTNSNAAGKQLRK--ESQKDRKNPLPPSVGVVDKKEETQPPVALKKEGIRRVGRRPDQQLQGEGKIIDRRP-ERRPPR 134

***S. kowalevskii*** 59 QVNSKKEPLQPSNTR--KEDSTRETLNKPPRRQQQQQQQQQPQQQDGDNSRRQGNRSTFNSFDIENKRPDNRR-NRRPFT 135

***A. planci*** 47 --KAEAEKKEPEPQQ-------------KPKAEETRQEPVEPRRSGGRGGNRGSSARQGG-------------eNRRPPR 98

***D. melanogaster*** 50 ATKTTANKVANSNNK--ANAGSNIGGPNAKKPNQAEKENKPNNALNKTDGKKFTPSADNKQQQFNNNASSNYK-----QQ 122

***S. mimosarum***  53 G--KSVS-SAGLPKK--AKENTAVNTPKHSETTEPS-NKTKA-KPFTDRTSKNTRNF-EDVKDVED---------RKNFR 115

***C. sculpturatus*** 54 V--TTTKVTKDIPTK--GKLNS-----------EPALHKTKSTKPGTDRTVRFSNNSQEDSKETEE---------RRNRR 109

***O. sinensis*** 52 NIKSTKKPVAPSPQE--SKSKAPEQSSQRRDVVESNRRESAPRGQNRGRGRSFRPNRETESKDSNEFG-DNET-ERRPPR 127

***T. circumcincta*** 64 VGKENSKPASGERGRggRGRGRGAGGPRPPRAEGEFAERFGEARGGRGGGPRGGGRGRGAPFRGRGGAANTFTdAPKEFQ 143

**E. granulosus** 70 APRNDRRGADQKASL--RDASGNIRGRAAPRGRGVSARNKAP---------KFG-------------------------- 112

***N. vectensis*** 47 --RPSVKDGKPQQTK---------------KDKEAVNEESKKDAKRGNRGPKDVRRENEKNTRLVNGPNDKSReDRENVP 109

***A. queenslandica*** 62 GGSKEEKGKKKGEKP--LEGNRKTEQQQQGKKKET-GERGPANKDKGPAGGE--RQRNNPPSGGGGGPTSGFKdNRRTNE 136

***H. sapiens*** 135 -------------ERRFEk--pl-----EEKGEGGEfs-------VDRPIIDRPiRGRGGLGRGR-ggrGRGMG-rgdGF 185

***S. kowalevskii*** 136 --PRDDTEGTPT-QTTPQ--------QVDFRNERDS-----RE----RSERG---RGFRGRGRGR----GGYFG----GR 184

***A. planci*** 99 --ERRDQDTSENqENHAP---------VEFRSEKSD-----RPvYRGAEQGDRPpRGERGRGRGR---gRGRVG-fagGR 158

***D. melanogaster*** 123 gaPRQGGGANRTrEFGSG--------QGQGQGQGGQ---qqRSvNFRQQNGNAEtREQRNNRRNV----RENVG-apdGQ 186

***S. mimosarum***  116 --NRDEHPPLSS-DVRE---------R-DGESRRG------RG-GF---------RG-RGRSRGR----GNFFG----NF 157

***C. sculpturatus*** 110 --NRDDK--LFS-DIRES--------R-DGENRRGG-----RG-GFSTTGGGGNmRGSRGRGRGR----GGNFG-----F 160

***O. sinensis*** 128 --RRDDF---SS-DYRRE--------RSDGFNRSSG-----RSdGFGRPEGGSG--KSEGFSGGR----SEGFG-----F 177

***T. circumcincta*** 144 ---------SSAdEVPSE--mpp----AEFHGERRG-------------RGGRG--AFNGERRGR--------Gaf---- 181

**E. granulosus** --------------------------------------------------------------------------------

***N. vectensis*** 110 --PRKGGFNRSReDGGQEggdspv--KVEQGGDRGG---------FGSRGGNRGaRGTGGRGRGG-------FG---aNR 166

***A. queenslandica*** 137 asDREGRPPRQQqQYRNRgrlpskenQESGGDEGGDdsrqtRQrWNNRERGDR--QGERGGERGGdtggEKRFNpfrtNR 214

***H. sapiens*** 186 DSR-----------------------------------------GK**R**E**F**D**R**H**SG**S**D**RS-----sfshySGLKHED**K**RG**G**S 219

***S. kowalevskii*** 185 DNR-----------------------------------------GK**R**E**F**E**R**H**SG**S**D**K-----------SSVKAQD**K**RD**G**A 212

***A. planci*** 159 DFR-----------------------------------------GK**R**E**F**D**R**H**SG**N**D**KS----------SSLKPQD**K**RE**G**A 187

***D. melanogaster*** 187 QSRp----------yrgpgg----gpgaggdrpqrqnrnydgqnRK**R**E**F**D**R**Q**SG**S**D**RT-----------GVKSID**K**RD**G**A 241

***S. mimosarum***  158 EGR-----------------------------------------PR**R**E**F**D**R**H**SG**S**D**K-----------SGLRPVE**K**RD**G**A 185

***C. sculpturatus*** 161 DSR-----------------------------------------GK**R**E**F**D**R**Q**SG**S**D**K-----------SGVKPVE**K**RE**G**A 188

***O. sinensis*** 178 NRNseegsfgrsdgfrgrgrrggrgggrgg-r----gvsfsdrgGK**R**E**F**E**R**H**SG**S**D**K-----------TGVKAVE**K**KE**G**G 241

***T. circumcincta*** 182 --R-----------------------------------------GG**R**Q**F**D**R**Q**SG**S**D**RT-----------GVRGDD**K**KG**G**Y 207

**E. granulosus** 113 --------------------------------------------GQ**R**V**F**D**R**H**SG**S**D**KT-----------GVKAVM**K**KD**G**H 137

***N. vectensis*** 167 G------------------------------------------gRK**R**E**F**E**R**R**SG**S**D**RSvpegpsvanmRSVKPQD**K**RE**G**G 204

***A. queenslandica*** 215 P-G---------------gg----ggggggf-----------rgGK**R**E**F**E**R**R**SG**S**D**KS-----------SVKAFD**K**RE**G**G 252

**II****cv**

***H. sapiens*** 220 **G**SH**NWG**TVKDELTESPKYiqkqisy-----------------nysdLDQSNVTEETPEG-----------EEHHPVADTE 271

***S. kowalevskii*** 213 **G**SH**NWG**NVRDDVKEATNP----------------------------ELNTSAVSDDAEY--------ANKTGDEATEPKD 256

***A. planci*** 188 **G**SH**NWG**TAKDDMRDLNTS------------------------nltdEAETPDYSGQPLD--adaqaKETTEDGEGKEGEA 241

***D. melanogaster*** 242 **G**SH**NWG**SVKEAIDDVNKN-----------------------------------ESETNV---------TNAEGGAKADES 277

***S. mimosarum***  186 **G**PG**NWG**DLKNEFNSRRREaestwmdeefdasgdkqndscwkeilpaETEMPAVSHNEL--taekcgDTTVPESMDSKENE 263

***C. sculpturatus*** 189 **G**AH**NWG**TVKDDLE---------------------------------VQLTPLSDENPE--igersgDEIVPEQNDIKETE 233

***O. sinensis*** 242 **G**SH**NWG**TMKDDMDST------------------------------nSREQLKETETQEWsaqaedaENQDPNETTAETTE 291

***T. circumcincta*** 208 **G**RG**NWG**DVKDELAGET--------------------------------EPIVVPEEPEV---------------PREKTA 240

**E. granulosus** 138 **G**TG**NWG**TLEDELEAQM------------------------------AEVVPEPTTEEEA--------PAEEVKSAESTPT 179

***N. vectensis*** 205 **G**QF**NWG**NPADTEEEGVVY----------------------------NQEKPDVEGSPEP-------GSPGSPKPDAEEGQ 249

***A. queenslandica*** 253 **G**AH**NWG**TVKDDMNTNEWAtdeq-------------------eaatdNNATELNEENTN--dqsevvEGEGETESKVAPGS 311

**III****cv**

**II****cv**

***H. sapiens*** 272 NKENEVEE-VKEEGPKEMT**L**DEWKAIQNKDRAKVE--FNIRKPNEGADG------------------------------- 317

***S. kowalevskii*** 257 GL-NEEEIaAKEAEAKQMS**L**DEYKAQVIMEREKLAnkFNFRKAGEGEDQA------------------------------ 305

***A. planci*** 242 AEEGEEESpKEEEGTRELT**L**DEYKEQLSKERSKAQ--FNVRRAGEGEDTS------------------------------ 289

***D. melanogaster*** 278 GTEPQNEQaTAEEEAKELTLDEWKAQQGQ-RIKPT--FNIRKAGEGEDTT------------------------------ 324

***S. mimosarum***  264 P--VTEE--RPEEGLREMT**L**DEWKRQQEAKRAVPK--YNLRKPGEGEDGN------------------------------ 307

***C. sculpturatus*** 234 LGIEGVK--ITNEANNEMT**L**DEWKKQQEGKRSKAS--FNLRKAGEGEDSN------------------------------ 279

***O. sinensis*** 292 AN-PEVP--DEEQIVKQMT**L**DEWKALEKKNRVKTE--FNIRKPNEGVDES------------------------------ 336

***T. circumcincta*** 241 EELAQEAL--EAEYAKQKT**L**KEY--IDSQKKEAPK--FNVRKAGEGEE-------------------------------- 282

**E. granulosus** 180 EQIQEPV--DERP--KTLT**L**REYREQLRASKAKVQ--LTTKGERRPNDGKnvfsdmvevhkskplepkvelvledkpqie 253

***N. vectensis*** 250 EQNEEGDA-EKEEEQKEMS**L**EEWKELQNKYRAKMS--FELRKPGEGEKKN------------------------------ 296

***A. queenslandica*** 312 EEKEGEEEeEEKE-TPQFT**L**DEYKAMQDKKRTVPL--HKERRPGENEDQT------------------------------ 358

**III****cv**

***H. sapiens*** 318 QWKKGFVLh------------------------------KSKSEEAHAEDSVMDHHF------RKPANDIT-SQLEINFG 360

***S. kowalevskii*** 306 QWKKTYVL-----------------------------KNKPDSKYDKAA---------DDKFVREKISKPT-VDIEIKFA 346

***A. planci*** 290 QWDRTVALk-----------------------------KQQKSEEEKPVVTAEHPHK-----DRSNKAQLV--DITISFN 333

***D. melanogaster*** 325 QWKKMVVL-----------------------------TSNKKKENDSEEELEYDPAL---YPQRVGRQQRV-LDIQFNFN 371

***S. mimosarum***  308 QWKKLYML------------------------------KKKAKEDDDEEDEDDDEDVdDEEFARRGRHRQL-VDIQINFN 356

***C. sculpturatus*** 280 QWKKFTVL-----------------------------TSKKFEEYDDEEEEEESED--DDEFVKRGRQKQV-VDIKINFA 327

***O. sinensis*** 337 QWKKTYVL-------------------------------KKKESDDEDEDEDEDEGYeDEP--RRGKNI-L-HNIKITFN 381

***T. circumcincta*** 283 --ENFGKL----------------------------vKLQKEVVVDKEEEEVSI-------IRREPREKAL--HIDIQFA 323

**E. granulosus** 254 EEAKTVVFefgrgrptprgragrpfgssefrgrgsgpRADRQSREELQAQPAEQTEQqSQESPDSSEPSGQ--PRERSAP 331

***N. vectensis*** 297 QWKNTQVLq-------------------------------------KEEISDEHGVMyEEKIKTSGRVKKAvPGIQFDFV 339

***A. queenslandica*** 359 RWKNTFVL-----------------------------ERNEAEFFPGTREIV-------EKYKTSGREKTV-IESSFFFR 401

**IV****cv**

**III****cv**

***H. sapiens*** 361 DLGRPGRG---------GRGGRGG--------------------------------------------RGRGGRPNRGSr 387

***S. kowalevskii*** 347 DT-----------SRGGGRGA-GS--------GPGRRGGGRGGGGRGRRG--------GG--------PGRGNNNMKNQ- 389

***A. planci*** 334 ESDRQSGE---srGGRGGRGGRGGrgg----rGGRGGSGGARDFDRDRERdrdrdateNDrgyt---pRGGGRGGGFGGr 403

***D. melanogaster*** 372 DG-RRGGP-----GGFGGRGGRGGprp-----------GGFGGGPRSEGGnrdggnreGGrdnr----EGGNRGPRDGQq 430

***S. mimosarum***  357 DS-RR--------GRGRGRGVRGV--------GPRSGGGSMGNKDSSREErremgssvGN--------RSFGRGSAKV-- 409

***C. sculpturatus*** 328 DS-RR--------ARGRGKA------------------------------------------------RGSGRGIMKGG- 349

***O. sinensis*** 382 DSPRR--------GRGGGRRGRGS--------GSERGGGERGT---------------GG--------RGGGRAISRAE- 421

***T. circumcincta*** 324 EP---------------NRGFRGD-----------------------------------------rppRGRGHAAVVDEv 347

**E. granulosus** 332 RGGRRGERvfrggSRGRGFIPRVGfna---erGRGRGGGPGGDRPSFRGGdrpsfr--GGe-------RGGGRGMRGGG- 398

***N. vectensis*** 340 NTEPKGDS------RGGGRGGRGGrpg----rGGRGGRGASGGRG-----------------------RGGGRGGFGGG- 385

***A. queenslandica*** 402 DPQRFTGG--rggGRGGGTGGRGGgrrpsderRGNFRRGDRDDRRDDRRGdrddrrgdRDerrgdrddRRGDRDDRRGD- 478

**V****cv**

**IV****cv**

***H. sapiens*** 388 t-------------------------------------------DKSSASAPDVD------------------------- 399

***S. kowalevskii*** 390 --------------------------------SSF-----------GGGAAPRMD------------------------- 401

***A. planci*** 404 g-------------------------------GSGrgrg----gGRGGGRGGGGGgggrggsrgggrgg--gassqapvm 446

***D. melanogaster*** 431 ------------------------------hnNEGggssaqnqrPPIDRRGPGNNqnnnqnsgpgpnkrferqqntap-- 478

***S. mimosarum***  410 --------------------------------KS------------GQQSAPRVD------------------------- 420

***C. sculpturatus*** 350 --------------------------------KS------------PEQSVPKVD------------------------- 360

***O. sinensis*** 422 --------------------------------KKG----------GPRESAPCMD------------------------- 434

***T. circumcincta*** 348 vvegtvhrfllnliprlmpfpllvpsklsfhcSSLqlsvisntfATTSARAH---------------------------- 399

**E. granulosus** 399 --------------------------------PRGggva-anvgGGGGFRRGRGAfdgpqearggtrgglraptntapsm 445

***N. vectensis*** 386 -------------------------------------------------AGPQGEghfe--------------------- 395

***A. queenslandica*** 479 --------------------------------RDDr------rgDRDDRRGDRDDrrgdrddrrg--gerrpprardrsn 518

**V****cv**

***H. sapiens*** 400 ----DPEAFPALA------------------- 408

***S. kowalevskii*** 402 ----DENDFPLLGK------------------ 411

***A. planci*** 447 ---tSEEDFPSLGSK----------------- 458

***D. melanogaster*** 479 -kvnDERQFPTLA------------------- 490

***S. mimosarum***  421 ----DWNDFPSLVTA----------------- 431

***C. sculpturatus*** 361 ----DWNDFPSLVTA----------------- 371

***O. sinensis*** 435 ----DENDFPRLVHTaa--------------- 447

***T. circumcincta*** --------------------------------

**E. granulosus** 446 ---dSDADFPALNSKvstiiheaaitcctsqv 474

***N. vectensis*** 396 ---iNSDEFPSLA------------------- 405

***A. queenslandica*** 519 fnveDEKDFPSLGTVsa--------------- 535

**V****cv**

**Supplementary Figure 1:** Comparison between SERBP1 protein homologues in 11 different species using COBALT. Sequence alignment was conducted to identify conserved regions in SERBP1 protein, prioritizing amino-acid identity. Human SERBP1 was used in reference. Conserved amino-acids (same amino-acid in at least seven species) are in red, highly conserved amino-acids (same amino-acid in all 11 species) are in red bold, RGG motifs are highlighted in yellow. Gray boxes are used to indicate highly conserved regions and cover the same residues as in Figures 1C and 1D.


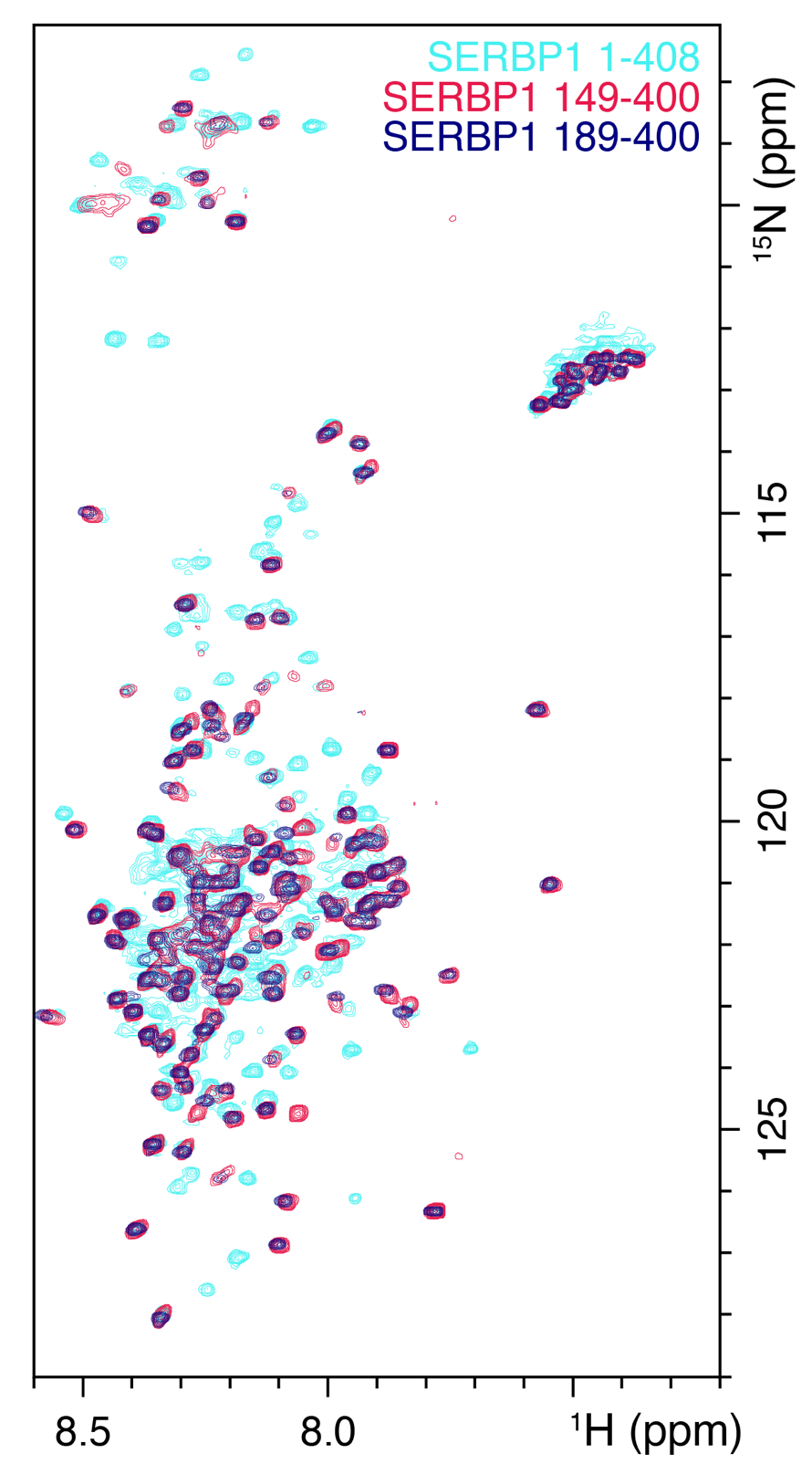


**Supplementary Figure 2:** Overlay of ^1^H,^15^N-HSQC spectra for the three SERBP1 constructs; 1-408, cyan, 149-400, red and 189-400, dark blue contours.


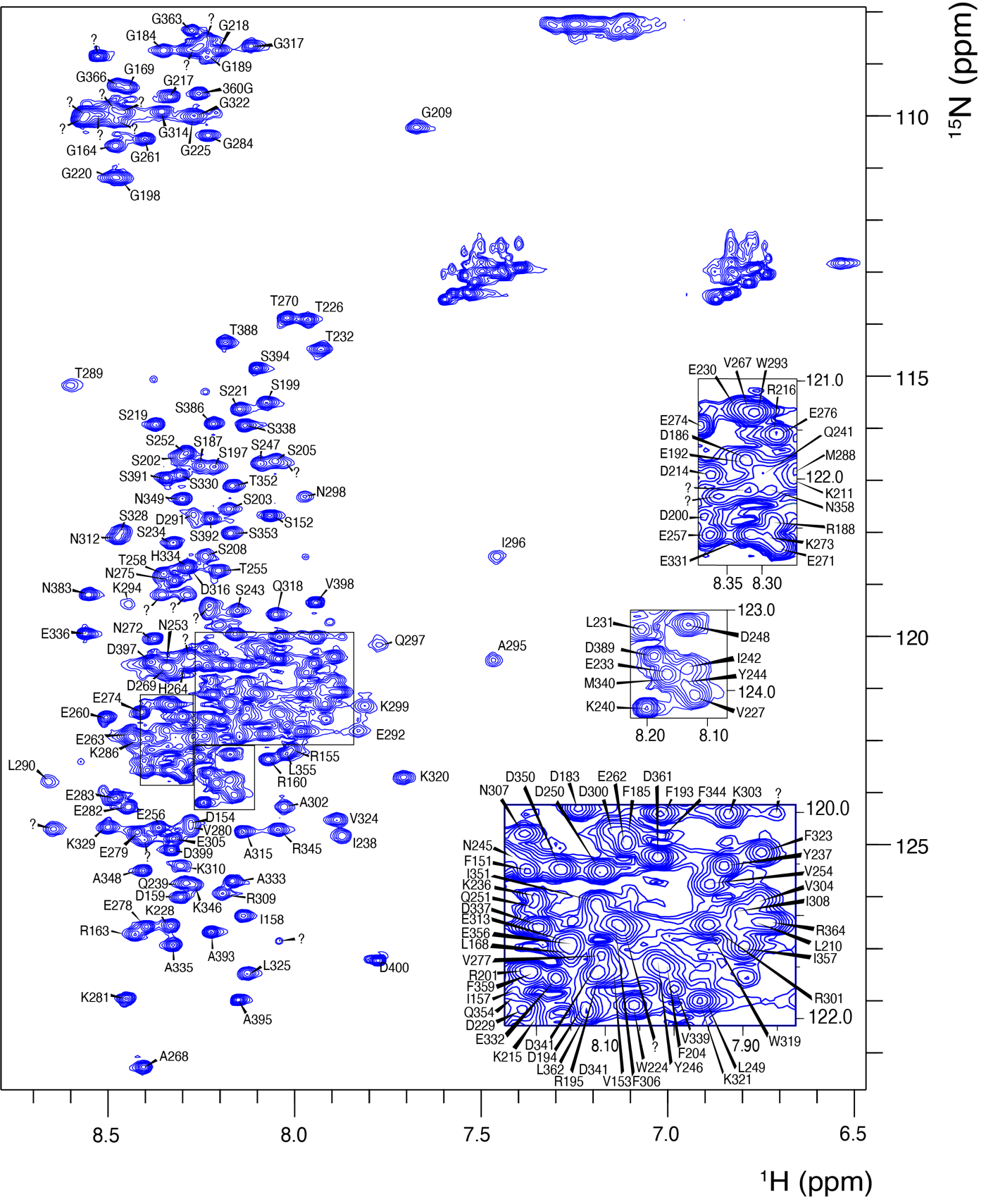


**Supplementary Figure 3:** Assigned ^1^H,^15^N-HSQC of SERBP1 149-400.


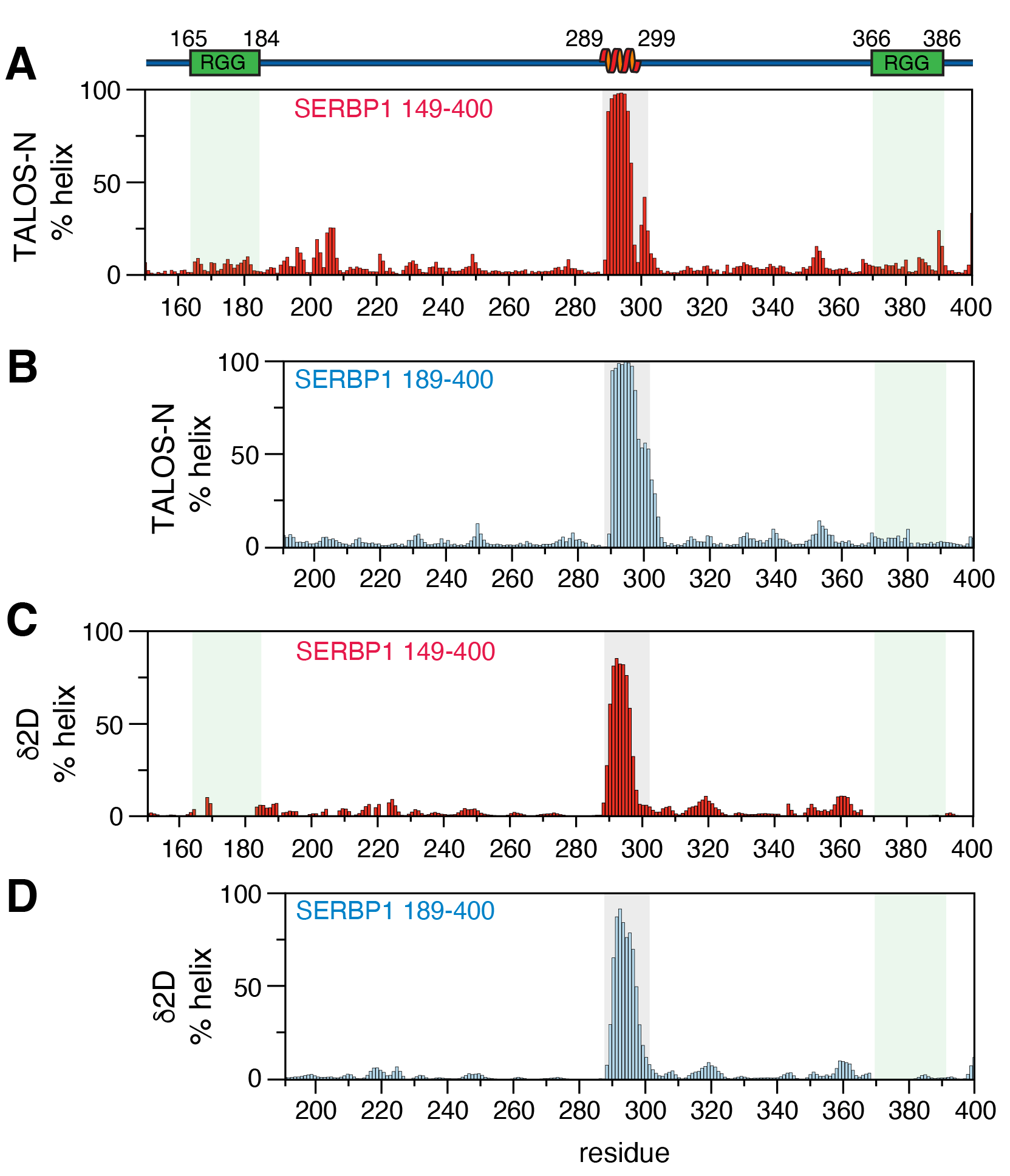


**Supplementary Figure 4:** Predicted secondary structure of SERBP1 149-400 and 189-400. Secondary structure propensities were calculated from backbone chemical shifts by the TALOS-N (top panels) and δ2D (bottom panels) algorithms for **(A, C)** SERBP1 149-400 and **(B, D)** SERBP1 189-400. Gray boxes outline residues 289-299, identified as having substantial α-helical propensity. A cartoon representation of the α-helix and relative position of the RGG boxes (green) is shown above the plots.


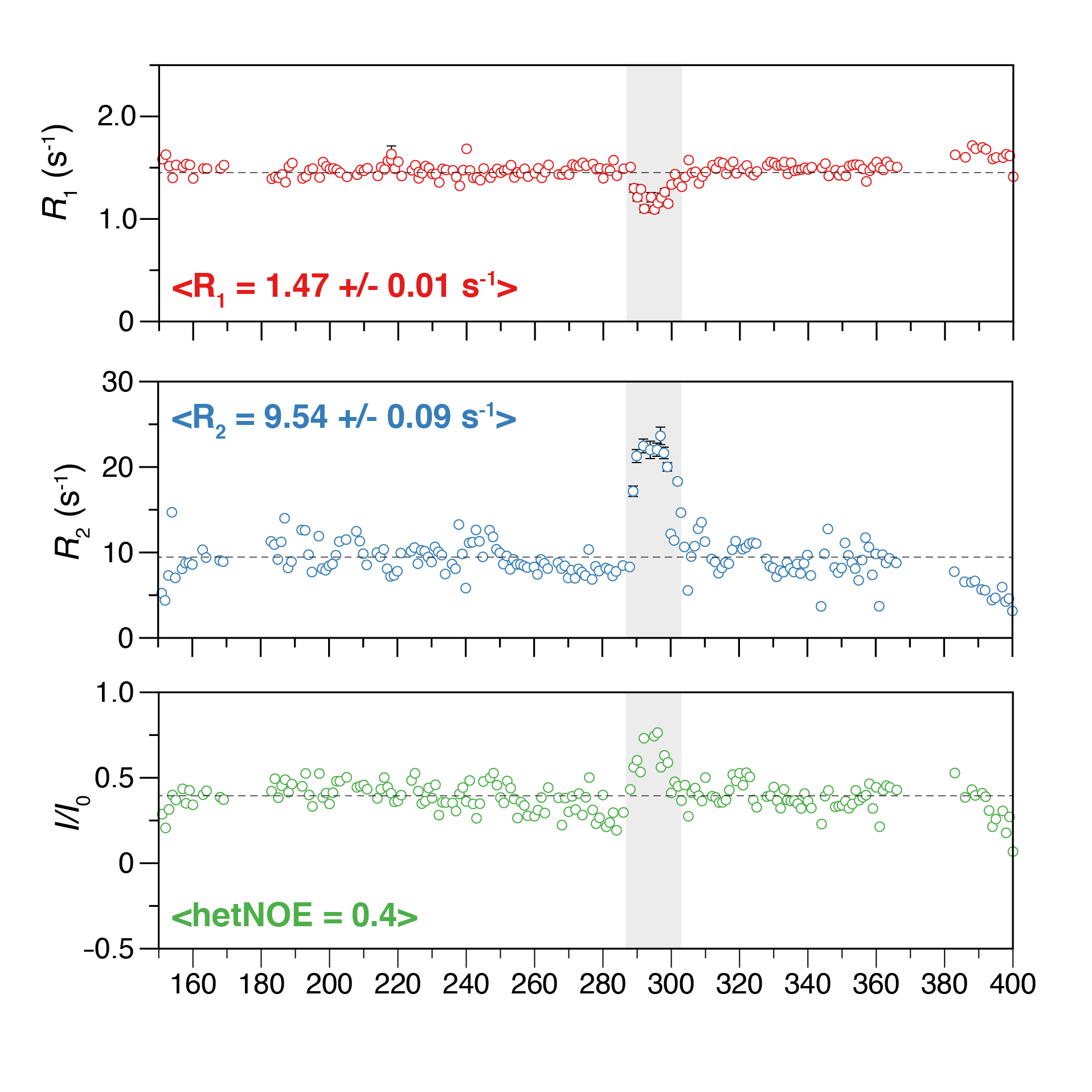


**Supplementary Figure 5:** ^15^N relaxation parameters of SERBP1 149-400. The ^15^N *R*_1_ and *R*_2_ rates, and the {^1^H}-^15^N heteronuclear NOE (top, middle and bottom panel, respectively) are plotted against the protein sequence. The dash lines represent the average value for each plot, also indicated in brackets. The gray boxes indicate a high propensity for a stable α-helix, as predicted in Figure 2A.


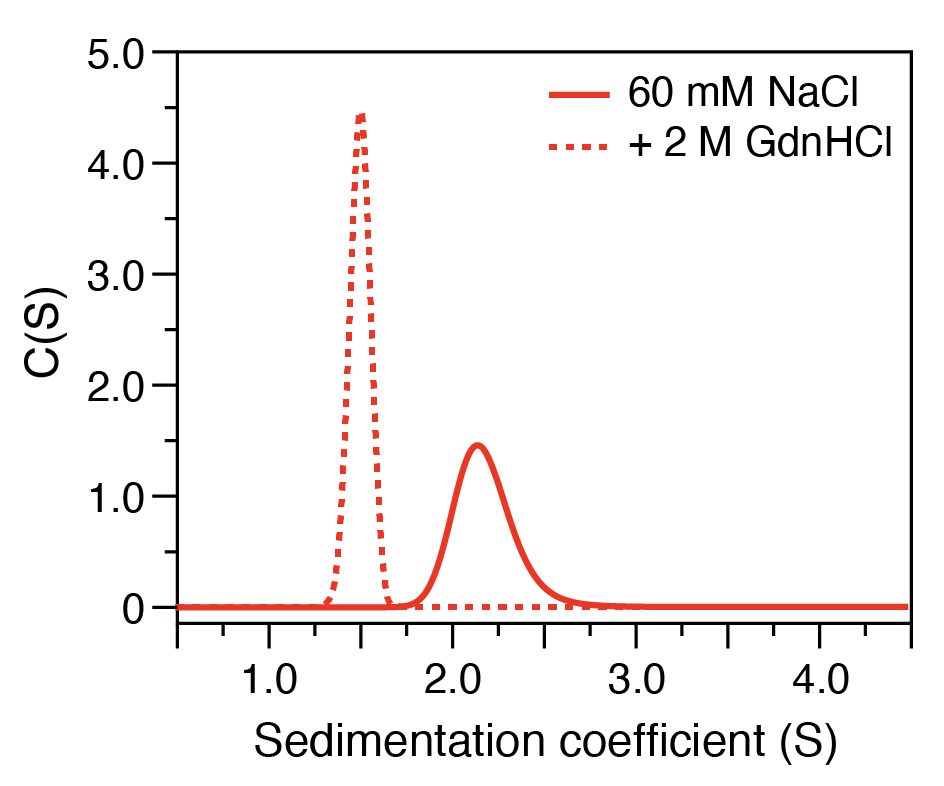


**Supplementary Figure 6:** AUC sedimentation coefficient profile of SERBP1 149-400. The protein was sedimented in absence (solid) and presence (dashed) of 2 M guanidinium chloride.


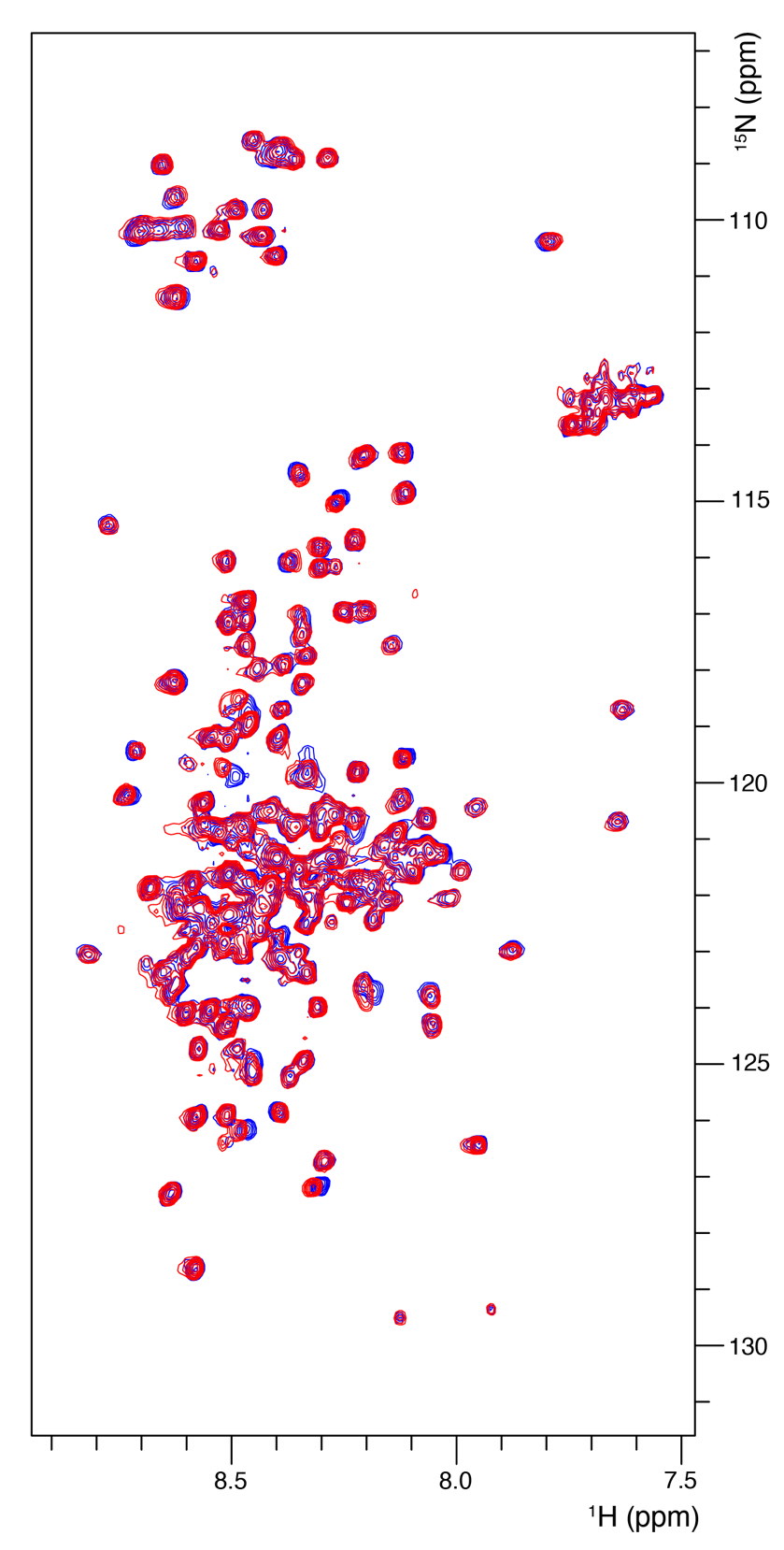


**Supplementary Figure 7:** NMR binding assays of SERBP1 189-400 with the AUAUAAA control RNA. Overlay of the ^1^H,^15^N-HSQC spectra of SERBP1 in absence (blue) and presence (red) of the 5’-AUAUAAA-3’ RNA at a protein:RNA ratio of 1:1.6.

**
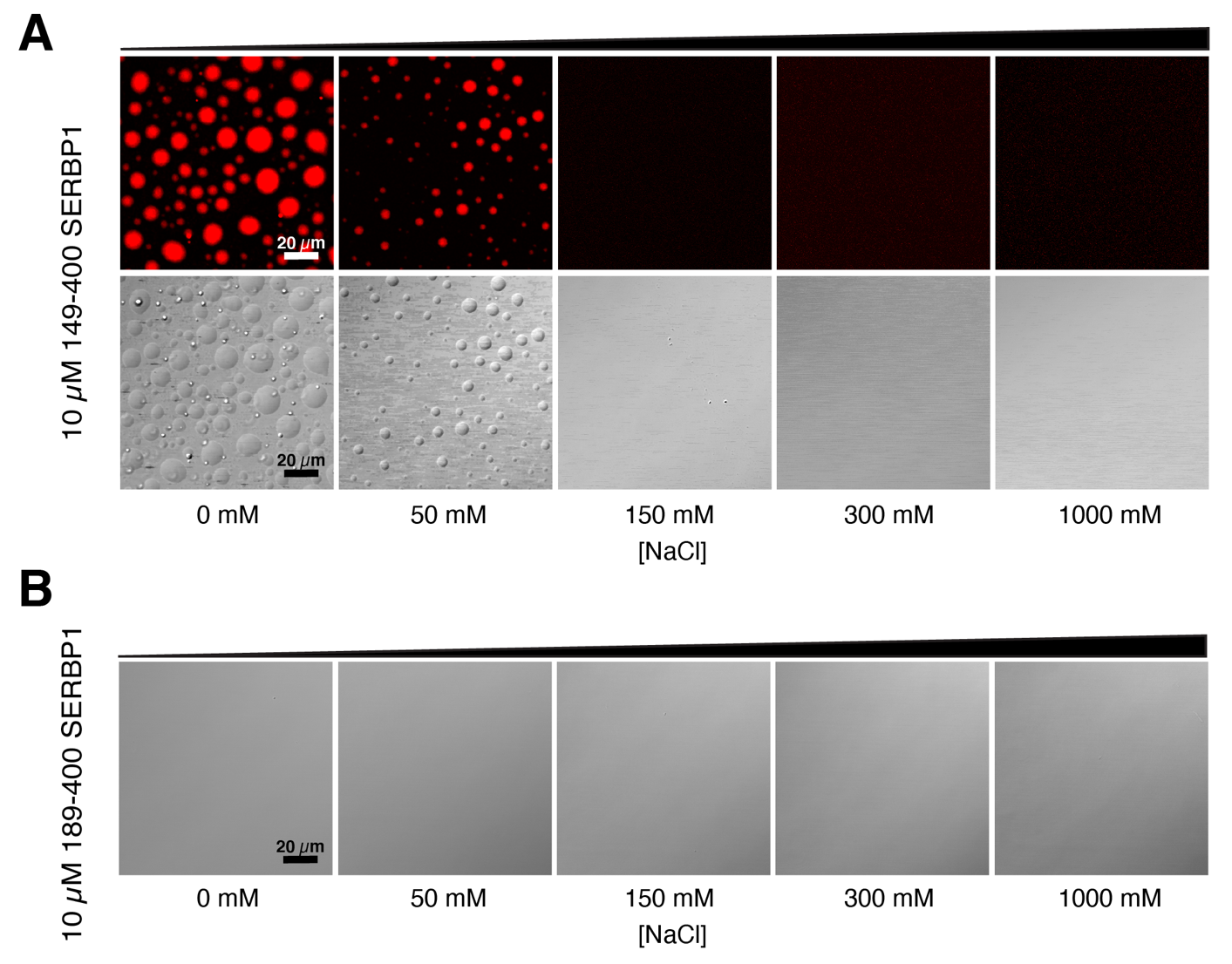
**

**Supplementary Figure 8:** SERBP149-400 and 189-400 phase separation in response to salt. **(A)** Effect of increasing NaCl concentration (0 to 1 M) on the LLPS propensity of 10 µM SERBP1 149-400 or **(B)** SERBP1 189-400. DyLight™ 650-labelled SERBP1 149-400 confirms the droplets contain protein.

**
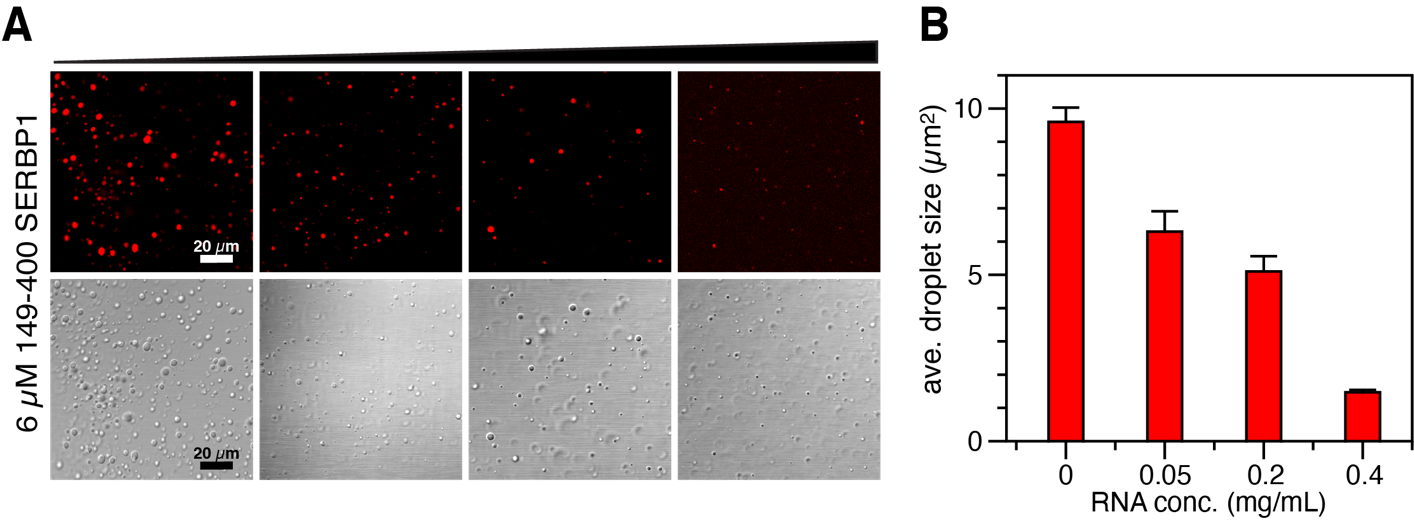
**

**Supplementary Figure 9:** Effect of RNA on phase separation of 6 µM SERBP1 149-400. Yeast RNA concentration series of 6 µM SERBP1 149-400, from 0 to 0.4 mg/mL of RNA. The average droplet sizes (µm^2^) are shown on the right panel bar plot.
